# Measuring naturally acquired immune responses to candidate malaria vaccine antigens in Ghanaian adults

**DOI:** 10.1186/1475-2875-10-168

**Published:** 2011-06-20

**Authors:** Daniel Dodoo, Michael R Hollingdale, Dorothy Anum, Kwadwo A Koram, Ben Gyan, Bartholomew D Akanmori, Josephine Ocran, Susan Adu-Amankwah, Harini Geneshan, Esteban Abot, Jennylyn Legano, Glenna Banania, Renato Sayo, Donald Brambilla, Sanjai Kumar, Denise L Doolan, William O Rogers, Judith Epstein, Thomas L Richie, Martha Sedegah

**Affiliations:** 1 Noguchi Memorial Institute for Medical Research, University of Ghana, Legon, Ghana; 2 Consultant to the US Military Malaria Vaccine Program, Naval Medical Research Center, Silver Spring, MD 20910, USA; 3 WHO Regional Office for Africa, Brazzaville, Congo Republic; 4 US Military Malaria Vaccine Program, Naval Medical Research Center, Silver Spring, MD 20910, USA; 5 RTI Rockville, Rockville, MD 20852, USA; 6 Center for Biologics Review and Research, Food and Drug Administration, Rockville, MD 20892, USA; 7 Naval Medical Research Unit #3, Cairo, Egypt; 8 Queensland Institute for Medical Research, Brisbane, QLD Australia

## Abstract

**Background:**

To prepare field sites for malaria vaccine trials, it is important to determine baseline antibody and T cell responses to candidate malaria vaccine antigens. Assessing T cell responses is especially challenging, given genetic restriction, low responses observed in endemic areas, their variability over time, potential suppression by parasitaemia and the intrinsic variability of the assays.

**Methods:**

In Part A of this study, antibody titres were measured in adults from urban and rural communities in Ghana to recombinant *Plasmodium falciparum *CSP, SSP2/TRAP, LSA1, EXP1, MSP1, MSP3 and EBA175 by ELISA, and to sporozoites and infected erythrocytes by IFA. Positive ELISA responses were determined using two methods. T cell responses to defined CD8 or CD4 T cell epitopes from CSP, SSP2/TRAP, LSA1 and EXP1 were measured by *ex vivo *IFN-γ ELISpot assays using HLA-matched Class I- and DR-restricted synthetic peptides. In Part B, the reproducibility of the ELISpot assay to CSP and AMA1 was measured by repeating assays of individual samples using peptide pools and low, medium or high stringency criteria for defining positive responses, and by comparing samples collected two weeks apart.

**Results:**

In Part A, positive antibody responses varied widely from 17%-100%, according to the antigen and statistical method, with blood stage antigens showing more frequent and higher magnitude responses. ELISA titres were higher in rural subjects, while IFA titres and the frequencies and magnitudes of e*x vivo *ELISpot activities were similar in both communities. DR-restricted peptides showed stronger responses than Class I-restricted peptides. In Part B, the most stringent statistical criteria gave the fewest, and the least stringent the most positive responses, with reproducibility slightly higher using the least stringent method when assays were repeated. Results varied significantly between the two-week time-points for many participants.

**Conclusions:**

All participants were positive for at least one malaria protein by ELISA, with results dependent on the criteria for positivity. Likewise, ELISpot responses varied among participants, but were relatively reproducible by the three methods tested, especially the least stringent, when assays were repeated. However, results often differed between samples taken two weeks apart, indicating significant biological variability over short intervals.

## Background

Naturally-acquired immune responses to *Plasmodium *spp. infection target a variety of pre-erythrocytic and blood stage antigens of the parasite[1]. When an endemic population demonstrates a degree of clinical or parasitological resistance, identifying an association between immunological recognition of a given antigen and resistance to malaria may indicate the antigen's potential value as a malaria vaccine candidate. Defining background responses is also useful for planning vaccine trials in endemic areas, due to the need to distinguish vaccine-induced responses from the baseline of naturally acquired responses once the vaccine is administered. Additionally, knowing this baseline and comparing immune responses post immunization with the responses obtained in malaria-naïves helps to assess whether naturally-acquired responses can be boosted by the candidate malaria vaccine.

Many studies have used immunofluorescence antibody assays (IFA) and enzyme linked immunosorbent assays (ELISA) to measure naturally acquired anti-malaria antibodies. In some cases, immunological studies have demonstrated an association between positive anti-merozoite[2] or anti-pre-erythrocytic[3] antibodies and incidence of malaria infection. IFA positivity has generally been defined by titres equal to or higher than the dilution of control sera not giving a positive immunofluorescence with the test antigen[4,5], while ELISA positivity has generally been defined as titres greater than the mean + 3 SD of the negative control samples ("classical approach"). It has been pointed out that there are problems associated with the classical approach when negative and positive samples are not well separated and the background levels of controls are variable; in this case, a latent class model may better estimate the proportion of positive samples [6]. However, it seems best suited to estimating the proportion of positive samples rather than identifying each sample as positive or negative, which is the objective of this study, and so was not used. Because the "the classical method" yielded many positive samples due to the small SD of negative controls, a second, more stringent method was also applied, in which a sample was deemed positive if it met criteria for the first method and simultaneously showed an optical density (OD) ≥ 0.5 at a dilution of >1/100.

Defining baseline, naturally acquired T cell immunity is more challenging, since activities of T cells measured using different assays in malaria endemic areas are low, vary over time[7-9] and are HLA-restricted[9]. Earlier studies, using proliferative or cytotoxic T cell responses to measure T-cell immunity, identified various epitopes within CSP [10-13], MSP1 [14-19], LSA1 SSP2/TRAP CSP [20-22] and AMA1[23,24] that induced recall responses in residents of endemic areas. In general, these T cell responses were short lived and did not clearly correlate with natural-transmission induced immunity defined as resistance to clinical malaria. Moreover, patent infections with *P. falciparum *appeared to suppress T cell responses[25]. Subsequently, *ex vivo *ELISpot assays were shown to be more sensitive than CTL assays [26,27]. However, ELISpot responses were also of low magnitude [9] and relatively unstable over time [7-9]. In studies in The Gambia and Kenya [28], positive ELISpot activities ranged from 28-34 sfc/m to SSP2/TRAP, 32-60% of volunteers responded, and responses differed when measured one year apart [9]. With MSP-1, responses differed when measured three weeks apart[8]. Such studies relied on a single assay per sample, albeit often using replicate wells; so far there have been no studies reporting whether such single assays reproducibly represent T cell immunity and thus whether differences measured at different time points might reflect intrinsic variation in the assay as much as changing T cell function.

ELISpot assays are generally defined as positive when a statistically significant difference is found between test samples and medium controls using the Student *t *test[29-31] or chi-square comparison[32], but often mean sfc/m induced by an antigen have been low and within the range of medium-only controls[29,33].

To assess baseline immune responses as well as their reproducibility, in preparation for vaccine trials in endemic areas of West Africa, healthy adult subjects were recruited from two sites in southern Ghana, the rural community of Mampong about 35 kilometres northeast of Accra, and the urban community of Legon, a northern suburb of Accra and site of the University of Ghana. In Part A of this study, IFA was used to measure antibodies to sporozoites and blood stage parasites, and ELISA was used to measure antibodies to the candidate recombinant protein vaccine antigens CSP, SSP2/TRAP, EXP1, LSA1, MSP1 and MSP3[10-24,34]. Antibody assays were defined as positive using the two approaches described above. *Ex vivo *interferon-g (IFN-γ) ELISpot assays were also performed, using fresh PBMC stimulated with HLA-matched peptides from the candidate antigens CSP, SSP2/TRAP, EXP1, LSA1 and LSA3. In the ELISpot assays, positive activity was determined by requiring a statistically significant difference between test sample (done in quadruplicates) and medium control using Student's *t *test, plus a 2-fold greater value than medium controls and at least a 10 sfc/m difference between test sample and medium controls.

Part B of the study was conducted two and a half years later using a different set of 12 volunteers, to assess the intrinsic reproducibility of the ELISpot assay. Cell samples were drawn from each study volunteer approximately two weeks apart and each sample was assayed three times on three different days to measure assay variability. Reproducibility of the assay was defined as the proportion of assays yielding either all positive or all negative results in the three replicate assays from a single sample time point while reproducibility over time was defined by comparing results from two time points.

Peptide pools spanning the full length of the test antigens CSP and AMA1 were used for stimulation in Part B rather than HLA-matched peptides, as a more straightforward approach that avoided having to perform HLA typing for each volunteer. Three statistical methods using different cut-off criteria of varying stringency were used to define positive responses to see which provided optimal results; (1) Least stringent: a greater than 20 sfc/m difference between peptide stimulated samples and medium controls; (2) Medium stringent (same as Part A): a significant difference on Student's *t *test comparing test samples and medium controls, at least two-fold difference comparing test samples and medium controls, and at least a 10 sfc/m difference between test samples and medium controls; and (3) Most stringent: based on criteria established for chronically HIV-infected subjects where a positive response was defined as a minimum of 55 sfc/m and a 4-fold difference over the medium controls [35]. Volunteers who developed positive ELISpot activities to one or more peptide pools were designated as positive responders.

## Methods

### Ethics

This study was conducted according to a human use protocol "Quality Control of Immunological Reagents and Validation of Improvements to Immunological Assays in Support of Malaria Vaccine Trials," approved by Institutional Review Boards at the Noguchi Memorial Institute for Medical Research (NMIMR) and the Naval Medical Research Center (NMRC). NMIMR holds a United States Government Federal Wide Assurance (FWA00001824) from the Office for Human Research Protections, as does NMRC (FWA00000152). NMRC also holds a Department of the Navy Addendum to the FWA for human subject protections. The protocol was conducted in accordance with all federal regulations regarding the protection of human participants in research including The Nuremberg Code, The Belmont Report, The Helsinki Declaration (1964 and as subsequently amended), 32 CFR 219 (The Common Rule) and all regulations pertinent to the Department of Defense, the Department of the Navy, the Bureau of Medicine and Surgery of the United States Navy and internal NMRC policies for human subject protections and responsible conduct of research. All NMRC and NMIMR personnel contributing to or performing human research efforts were certified as having completed human research ethics education curricula and training under the direction of the NMRC Office of Research Administration (ORA) and Human Subjects Protections Program (HSPP).

### Study site

Mampong town with a total population of approximately 8000 is located in the Akuapem North district in the Eastern Region of Ghana about 35 km northeast of Accra. It lies on the Akuapem-Togo mountains range, with an elevation ranging between 381 m and 487.7 m above sea level. The spoken language in Mampong is Twi and the major occupation is farming, although a substantial proportion of individuals work in the district offices, hospital or schools. Health services are provided by the Ghana Health Service through the District Health Management Team, the Tetteh Quarshie Memorial Hospital, the Centre for Scientific Research into Plant Medicine, and also by some private clinics and traditional healers. The climate is cooler than in Accra and the area receives on average about 2000 mm of rainfall a year. Malaria transmission occurs throughout the year, but most malaria admissions at the district hospital occur during the months of April to November. Among 21 residents of Mampong sampled for a baseline study of immune responses in September 2002, a third (7/21) had peripheral *P. falciparum *parasitemia detected by thick film examination. Malaria is the leading cause of pediatric admissions and outpatient attendance at the district hospital.

The Legon area is approximately 10 km north from the centre of the capital city, Accra. It is well settled and home to the University of Ghana that has a large student population of over 20,000. Accra and its environs lie on a relatively flat coastal plain with the highest elevation being less than 100 m above sea level. Rainfall is less intense and less frequent than in Mampong. The average annual rainfall is below 1000 mm with more than half falling between April and June. Malaria transmission follows the pattern of rainfall and most malaria admissions to the health facilities occur between May and August. Malaria transmission in this urbanized and settled area occurs mainly along the peri-urban fringes that have suitable breeding sites for the anopheles vector in uncompleted buildings and excavated areas. In a survey of 20 individuals for a baseline study of immune responses, none were positive by thick blood film examination, and in a recent (May 2010) screen of 111 volunteers for another study only one was positive by thick film examination.

The study is based on the rationale that transmission and therefore immunity differs between Mampong and Legon. Part A of this study was conducted on blood samples drawn in April 2005 and Part B on samples drawn in October 2007.

### Participants

Eligibility criteria for the study were the following: age 18-55 years; males or females who were not pregnant or nursing; normal screening medical history and physical examination; hemoglobin >10 g/dL; absence of known immunodeficiency (> 400 CD4 + T cells/μL); and negative hepatitis B and C serology.

### Sample collection

Blood was obtained by venipuncture and transferred into heparinized flasks using aseptic techniques. All blood samples were transported in cool boxes to the Department of Immunology of the NMIMR for analysis. Peripheral blood mononuclear cells (PBMCs) were separated from the blood samples by density centrifugation, washed and counted. Plasma obtained from the same separation procedure was distributed into aliquots and stored at -80°C for assessment of antibody levels. Blood smears were stained with Giemsa and examined for the presence of *Plasmodium *species using light microscopy.

### Control blood samples

Sera from malaria-naïve adults from Denmark and the USA were also used in ELISA and IFA assays.

### HLA typing

For each participant in Part A, low-moderate resolution HLA typing for HLA-A, HLA-B, and HLA-DR was conducted using the ABDR SSP Unitray system (Pel-Freez, Brown Deer, WI) according to the manufacturer's instructions.

### Synthetic peptides and peptide pools

For Part A, thirty-two short peptides (8-11 amino acids) with known restriction by HLA-A or HLA-B alleles and 25 long peptides (15-34 amino acids) that were DR epitopes from CSP, SSP2/TRAP, EXP1, LSA1 and LSA3 [36,37] were synthesized commercially (Chiron Technologies, Clayton, Victoria, Australia) and were used to assess IFN-γ ELISpot responses. Positive control peptides for CD8 + T cell-mediated responses consisted of HLA-A2.1 or -A3 restricted peptides derived from CMV or influenza virus. Purified protein derivative (PPD) (Statens Serum Institute, Copenhagen) was used as a positive control for CD4+ T cell responses.

For Part B, ELISpot assays used peptide pools of 15 amino acid (aa) synthetic peptides overlapping by 11 aa (Chiron Technologies, Clayton, Victoria, Australia) covering full length CSP and AMA1(>80% purity). These were combined into nine pools for CSP (Cp1-Cp9) containing 3-12 peptides per pool and 12 pools for AMA1 (Ap1-Ap12) containing 10-12 peptides per pool [31].

### Enzyme-linked immunosorbent assay (ELISA)

The *P. falciparum *recombinant proteins used in the ELISA assays; CSP, SSP2/TRAP, LSA1, EXP1, MSP1, MSP3, EBA175 have been previously described [38-43]. Stock solutions of *P. falciparum *recombinant proteins were diluted in phosphate buffered saline, pH 7.2, to the optimal concentration of each antigen (0.1-4 μg/ml). ELISA was performed as previously described[44] using quadruplicates of serum diluted two-fold from 1/50 to 1/5120. The endpoint titre of antibodies was defined as the calculated serum dilution yielding an optical density of 0.5 in the assay.

#### Analysis of positive end points

To determine whether the ELISA activity was defined as positive, two methods were used. The first was an OD that was above the mean of control negative donors (Danish volunteers with no previous exposure to malaria) + 3 standard deviations (SD). Since these differences were often small, a second quantitative method was applied: a positive activity was defined as an OD ≥ 0.5 at a dilution of >1/100 *and *greater activity than control sera + 3 SD. The numbers of total positive ELISA activities per volunteer was determined in urban and rural volunteers using both methods.

### Immunofluorescent Antibody Assay (IFA)

*P. falciparum *(strain 3D7) sporozoite-specific antibodies were assayed by immunofluorescent staining of air-dried *P. falciparum *sporozoites, and *P. falciparum *(strain 3D7)-infected erythrocytes cultured *in vitro*, as described previously[45].

### *Ex vivo *IFN-γ ELISpot Assays

#### Part A

ELISpot assays were performed as previously described (42) using freshly isolated PBMC in quadruplicate using 400,000 cells/well. Each volunteer's PBMC were tested against 8-11-mer HLA Class I binding peptides, or long 15-34-mer HLA Class II binding peptides that matched the volunteer's HLA type (10 μg/mL). To reduce the number of ELISpot assays some peptides were combined into mixtures: long peptides: CSP D44 + D49, D46 + D47; SSP2/TRAP, D51-D59; LSA1 D64-D68; LSA3, D70 + D72; EXP1 D60 + D61 (6 peptide mixtures + 3 single peptides = 9 stimulants); short peptides: CSP D2 + D3, D4 + D5, SSP2/TRAP D12-D14, LSA1 D23-D26, LSA3 D31-35, EXP1 D18-D21. (6 peptide mixtures + 12 single peptides = 18 stimulants). PPD was used as a positive control for PBMC biomarkers[46], and the negative control was medium only as used by others (for example [47]). CEF peptides (which include epitopes from cytomegalovirus, Epstein-Barr virus and influenza virus recognized by CD8+ T cells) were included in assays as positive controls.

#### Statistical analysis

To identify positive ELISpot activities, differences between means of peptide-stimulated and non-stimulated (medium only controls) were compared as used in current NMRC vaccine trials based on previously described methods[48]. The assay was considered positive if there was (1) a statistically significant difference between the number of spot forming cells in triplicate or quadruplicate test wells and triplicate or quadruplicate control wells (Student's two tailed *t*-test), plus (2) at least a doubling of spot forming cells in test wells relative to control wells, and (3) a difference of at least 10 spots between test and control wells (modified from ≥ five spots as previously described[48]. The average of medium controls was subtracted from test samples and reported as adjusted sfc/m PBMC. Any well that contributed more than 50% of the standard deviation of the quadruplicate and which was either three-fold greater or lower than the mean of the remaining cells were discarded as an outlier. The volunteer was designated as a responder at a single time point if his/her PBMC tested positive against at least one of the peptides for a given antigen.

#### Part B

ELISpot assays used pools (at 10 μg/mL) of synthetic 15-mer peptides spanning full length CSP or AMA-1 based on the premise that at least one peptide within a pool should have the correct sequence recognized by any HLA within the study population. Twelve volunteers were each sampled up to four times; samples for CSP were drawn on day 0 and day 14, and samples for AMA1 were drawn on day 9 and day 21. Thus the two sample time-points selected to control short term reproducibility were approximately two weeks apart for each antigen. Frozen PBMC aliquots from each sample were then tested in three separate ELISpot assays (1, 2 and 3) each performed on different days, and each sample from each assay was performed in triplicate. Each sample was tested with either 9 CSP peptides/peptide pools (Cp1 - Cp9) or 12 AMA1 peptides/peptide pools (Ap1 - Ap12) at 10 μg/mL. Positive controls were ConA (mitogen for cell viability) and CEF (a pool of peptides from cytomegalovirus, Epstein Barr virus and influenza virus that specifically stimulate CD8+ T cells).

#### Statistical analysis

To distinguish specific ELISpot responses, ELISpot activities using CSP or AMA1 peptide pools were compared with medium only controls and specific activity was determined using three methods of increasing stringency. Method 1: average of peptide pool stimulated activity minus that of the average of medium controls, with an arbitrary cut off of 20 sfc/m. Method 2: that used in Part A. Method 3: the cut off described for HIV *gag *protein[8], namely a median of >55 sfc/m and at least four-fold greater than the median of medium-only controls. As in Part A, a volunteer was designated as a responder at a given time point if his/her PBMC tested positive against at least one of the peptide pools for a given antigen.

## Results

### Participant flow

In Part A, 35 healthy adult volunteers recruited in Legon (n = 14) and in Mampong (n = 21) met the eligibility criteria and passed clinical and laboratory screening and were HLA-typed. Sera were tested in IFA and ELISA. PBMC were collected from 30 of these volunteers (Legon n = 13, Mampong n = 17) and fresh PBMC were stimulated with Class I and Class II peptides in ELISpot assays. Volunteer identification numbers, parasitaemia status and the tests performed are shown in Additional File 1 for the two communities.

In Part B, 12 new urban volunteers donated PBMC on four different days. The PBMC were frozen and tested in ELISpot assays using pools of overlapping peptides spanning the length of CSP and AMA1. Of these 12, seven were included in the analysis of reproducibility. Five were excluded because sampling was incomplete or else there were insufficient PBMCs to complete the analysis. Two of the seven volunteers (v1001 and v1041) had blood drawn only on two of the four sample days but were included for the antigen for which the sample set was complete.

### Antibody responses

#### IFA titres of urban and rural volunteers

**Figure 1:**
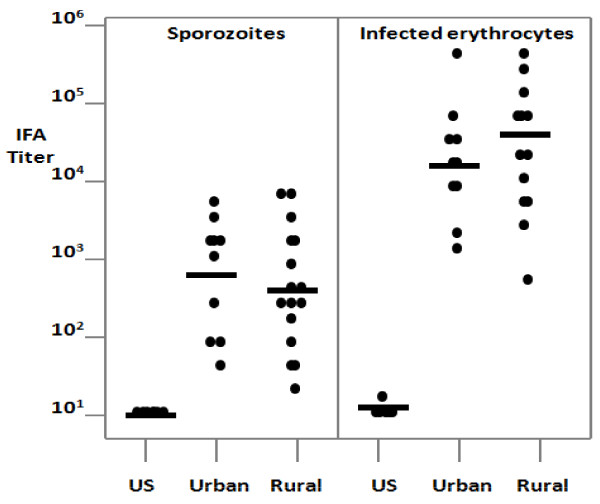
**IFA titers of sera from volunteers in Accra or Mampong compared with naïve controls from the US**. Horizontal bars represent geomean titers and filled circles represent individual titers.

The geometric mean anti-sporozoite IFA titres were 860 and 580 for residents of Accra and Mampong, respectively (Figure 1). The difference in titres between the two sites was not significant (P = 0.44, two tailed t-test on log transformed data). Geometric mean anti-infected erythrocyte titres were 22,000 and 55,000 in samples from Accra and Mampong, respectively, which likewise were not significantly different although there was a trend toward higher responses in the rural community (P = 0.081, two tailed t-test, log transformed). Sera from malaria-naive North American subjects did not react in IFA at dilutions greater than 1:10 (sporozoites) or 1:20 (infected erythrocytes).

#### ELISA titres of urban and rural volunteers

**Figure 2:**
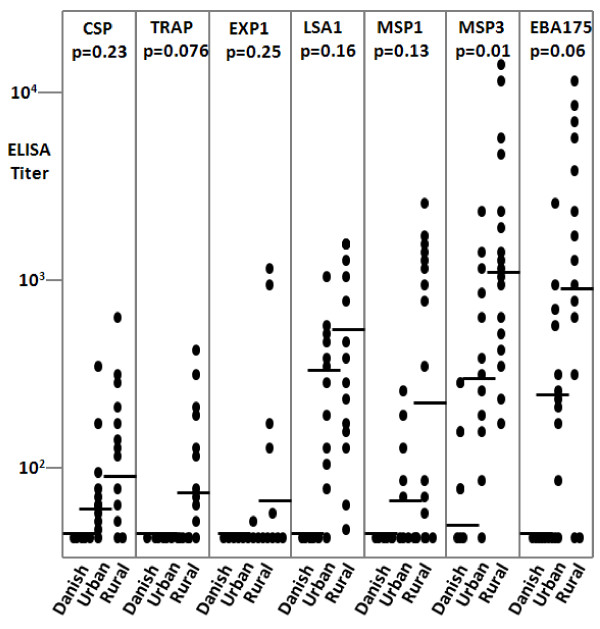
**ELISA titers of sera from volunteers in Accra or Mampong compared with naïve controls from Denmark**. Horizontal bars represent geometric mean titers and filled circles represent individual titers. P values are the differences in activity for each antigen between urban and rural Ghana populations; the only significant difference was for MSP3.

Activity was defined as the calculated serum dilution yielding an optical density of 0.5 in the assay[49]. The ELISA titres of 14 urban and 21 rural volunteers to seven *P. falciparum *candidate vaccine antigens are shown in Figure 2. While there are limitations to comparing titres to different antigens measured in different ELISA assays, group mean titres against the sporozoite antigens CSP and SSP2/TRAP were approximately ten-fold lower than those against the major blood stage antigens MSP1, MSP3, and EBA175, consistent with the lower IFA titres against sporozoites compared to infected erythrocytes. There was a trend for higher titres against all antigens in the Mampong subjects compared with those from Legon, although the difference was statistically significant only with MSP3.

The several antibody responses measured in each individual were moderately well correlated. In pair-wise correlations, log transformed ELISA titres to each of the four primarily erythrocytic stage antigens (MSP1, MSP3, EBA175, EXP-1) were correlated with each of the others (R = 0.5-0.7, p = 0.003-0.0001) and to the IFA titres against infected erythrocytes (R = 0.4-0.6, p = 0.03-0.0001). Similarly, ELISA titres against the three pre-erythrocytic stage antigens (CSP, SSP2/TRAP, LSA1) were significantly correlated with each other and with the sporozoite IFA titres (R = 0.5-0.6, p = 0.007-0.0005), except that titres against SSP2/TRAP were not significantly correlated either with titres to PfCSP or to sporozoites. Six of the volunteers from Mampong had *P. falciparum *parasitaemia on the day samples were collected. There was no significant difference in antibody titres in all ELISA assays between subjects with or without parasitaemia (p = 0.41 Mann Whitney U test two-tailed).

#### Definition and frequency of positive ELISA responses in urban and rural volunteers

##### Method 1

The mean ELISA activities from all the urban or rural volunteers were greater compared with the mean + 3 SD of the OD of control sera for at least one antigen (Table 1 and Additional File 2). Four erythrocytic stage antigens (EXP1, MSP1, MSP3 and EBA175), and two pre-erythrocytic antigens SSP2/TRAP and LSA-1 were frequently positive. A major exception was CSP where only 2/14 (14%) of urban and 11/21 (52%) of rural volunteers had positive responses. Urban volunteers were positive with fewer numbers of antigens than rural volunteers (4.9 and 5.9 antigens/volunteer respectively) and this difference was significant (p = 0.026, Mann-Whitney U test, 2 tailed).

**Table 1:** The frequency of positive ELISA activities conducted in urban and rural volunteers with 7 recombinant proteins representing some candidate vaccine antigens

Method			Antigens tested								
**1**	**Site**	**No. vol**	**CSP**	**TRAP**	**EXP1**	**LSA1**	**MSP1**	**MSP3**	**EBA**	**Total**	**Tot/v**

	**Urban**	14	2* (14%)	10 (71%)	11 (79%)	12 (86%)	11 (79%)	14 (100%)	8 (57%)	69	4.9

	**Rural**	21	11 (52%)	21 (100%)	19 (90%)	20 (95%)	17 (81%)	20 (95%)	16 (90%)	124	5.9

	**Total**	35	13 (37%)	31 (89%)	30 (86%)	32 (91%)	28 (80%)	34 (97%)	24 (86%)	192	5.5

**2**	**Urban**	14	0 (0%)	4 (29%)	5 (36%)	12 (86%)	10 (71%)	14 (100%)	2 (14%)	47	3.4

	**Rural**	21	5 (24%)	10 (48%)	10 (48%)	19 (90%)	14 (67%)	19 (90%)	5 (24%)	82	3.9

	**Total**	35	5 (14%)	14 (40%)	15 (43%)	31 (89%)	24 (69%)	33 (94%)	7 (20%)	129	3.7

Positive ELISA activities were defined using method 1 (OD that was above the mean of control negative donors (Danish volunteers with no previous exposure to malaria) + 3 SD) or method 2 (OD ≥ 0.5 at a dilution of >1/100 *and *greater activity than control sera + 3 SD). The Table shows the numbers of urban and rural volunteers that were positive with each antigen. The total numbers of antigens positive with urban and rural volunteers, and the frequency of positive antigens/volunteer (Tot/v) are shown in the two right columns. * Number of volunteers positive.

##### Method 2

When the more stringent second method was applied (Table 1 and Additional File 3), the numbers of urban and rural volunteers positive with CSP, SSP2/TRAP, EXP1 and EBA175 declined compared to the outcomes using the first method. However, all volunteers were positive with at least one malaria protein. The frequencies of positive assays with LSA1 and MSP3 remained similar, but slightly declined for MSP1 and declined more greatly for EXP1 and SSP2/TRAP. The greatest decline was with EBA175 and CSP; CSP was only positive with rural volunteers. The numbers of antigens recognized by urban and rural volunteers were 3.4 and 3.9 antigens/volunteer respectively and this difference was not significant (p = 0.5), in contrast to using method 1. The different outcome likely relates to the lower frequencies obtained with method 2 compared with method 1 for urban and rural volunteers (p = 0.02, p = 0.001 respectively), and this was largely attributable to differences with CSP, SSP2/TRAP, EXP1 and EBA175.

Six volunteers had malaria parasitaemia at the time the samples were taken but they appeared to recognize the same numbers of proteins as non-infected volunteers using method 1 and method 2 (Additional Files 2 and 3).

### Ex vivo IFN-γ ELISpot responses

#### Part A

##### Peptides used in Part A

Six mixtures and three single peptides (nine stimulants) of long DR-restricted and six mixtures and 12 single peptides of HLA A or B-restricted short peptides (18 stimulants) were used, and were numbered 1-27 (see Additional File 4 for sequences). Since this assay was based on matching the HLA of each volunteer with matched HLA A- or B- or DR peptides, not every volunteer was tested with every peptide. The combinations that were tested are shown in Additional File 5.

DR peptides: All volunteers were tested with five of the DR-binding peptides (all of these being mixtures) except one volunteer (v1340) who was not tested with LSA3. The remaining four DR stimulants (one mixture and three single peptides) were matched to varying numbers of volunteers based on predicted HLA A or B epitopes within their sequences. This resulted in 169 assays, 76 assays with 13 urban volunteers and 93 assays with 17 rural volunteers (Table 2, Additional File 5)

**Table 2:** Frequency of positive ELISpot assays conducted in urban and rural populations using long (LP) and short (SP) peptides

	No. assays		Total	No. positive assays/all assays		Total
**Pop**.	**LP**	**SP**	**LP+SP**	**LP**	**SP**	**LP+SP**

**Urban (n = 13)**	76	52	128	11/76 (14.5%)	3/52 (5.8%)	14/128 (10.9%)

**Rural (n = 17)**	93	44	137	22/93 (23.7%)	5/44 (11.4%)	27/137 (19.7%)

**Total**	169	96	265	33/169 (19.5%)	8/96 (8.3%)	41/265 (15.5%)

Six mixtures and 3 single peptides (9 stimulants) of long (DR-binding) or 6 mixtures and 12 single of short (HLA A or B-restricted) peptides (18 stimulants) were tested with HLA-matched urban and rural volunteers. The number of assays that were performed and the number of assays that were positive are shown and expressed as per cent of total assays. The sequences of the peptides, and how they were combined, are shown in Additional File 4.

HLA-A and B-restricted peptides: HLA matching peptides were available for 15 volunteers for CSP, 13 volunteers for SSP2/TRAP and LSA1, and 12 volunteers for EXP1 and LSA3, representing 17 of the 30 volunteers. These included matches for HLA-A01, -A02, -A02.1, -A03, -B07, -B35 and -B53. HLA matching for the A and B loci was based on HLA type, not supertype. This study did not have peptides matching the other HLA alleles identified in the study subjects. This resulted in 96 assays; 52 assays with 8 urban volunteers and 44 assays with 9 rural volunteers (Table 2, Additional File 5).

##### Frequency of positive assays

When all assays were analysed (Table 2), long DR-specific peptides elicited fewer responses in urban volunteers (11/76 assays, 14.5%) than rural volunteers (22/93 assays, 23.7%). Positive responses to DR-specific peptides were approximately twice as frequent as responses to short HLA class I peptides in both populations (urban 3/52 assays 5.8%, rural 5/44 assays 11.4%,) (Table 2). In total, there were 14/128 (10.9%) positive assays with urban volunteers and 27/137 (19.7%) positive assays with rural volunteers, suggesting that ELISpot responses to individual peptides or peptide pools were more frequent in rural than urban volunteers, with the caveat that a restricted number of HLA types were tested. The volunteer-peptide combinations are shown in Additional File 5.

##### Frequency of positive volunteers

Table 3 shows the numbers of urban and rural volunteers responding to at least one long or one short peptide. While urban volunteers had a slightly lower frequency of positive responders than rural volunteers to long peptides (urban: 6/13 volunteers, 46.2%; rural: 9/17 volunteers, 52.9%) and to short peptides (2/8 urban volunteers, 25%; 3/9 rural volunteers, 33.3%), taken together, urban and rural responders to both long and short peptides were similar (urban 8/13 volunteers, 61.5% of volunteers; rural 9/17 volunteers, 52.9% of volunteers). Therefore, while rural volunteers recognized more peptides than urban volunteers (Table 2), there was no difference in the frequency of volunteers who were positive to one or more peptides (Table 3).

**Table 3:** Frequency of volunteers with positive ELISpot responses conducted in 30 volunteers from urban and rural populations

	No. positive volunteers		
**Pop**.	**No. LP positive**	**No. SP positive**	**Pos. All peptides**

**Urban (n = 13)**	6/13 (46.2%)	2/8 (25.0%)	8/13 (61.5%)

**Rural (n = 17)**	9/17 (52.9%)	3/9 (33.3%)	9/17 (52.9%)

The numbers of urban or rural volunteers tested and the numbers positive for at least one assay with long or short peptides is shown for each group. While all volunteers were tested with at least one long DR peptide, only 8 urban and 9 rural volunteers were tested with short HLA A or B-matched peptides.

##### Magnitude of ELISpot responses to long DR-restricted peptides from each antigen

The volunteers recognized the 6 mixtures of DR-restricted long peptides but none of the single peptides (Table 4). One mixture (number 22) and the 3 single peptides were only tested with 8 and 6 HLA A and B matched volunteers respectively, and it is possible that testing more volunteers with these peptides may have revealed additional positive outcomes. Positive responses were obtained with peptides from all five malaria proteins tested: CSP, SSP2/TRAP, LSA1, LSA3 and EXP1. ELISpot responses ranged from 70 - 430 sfc/m, and were similar for urban and rural volunteers (p = 0.44 Mann Whitney U test two-tailed). LSA1 was most frequently recognized (11/30 volunteers, 37%), followed by SSP2/TRAP (8 volunteers, 27%), EXP1 (6 volunteers, 20%), LSA3 (4 volunteers, 14%) and CSP (3 volunteers, 10%).

**Table 4:** Individual urban and rural volunteers' fresh ELISpot activity with DR-matched long Class II-restricted peptides

		CSP		SSP2	LSA1	LSA3	EXP1
**Site**	**Vol**.	**D44,D49**	**D46,D47**	**D51-D59**	**D64-D68**	**D70,D72**	**D60,D61**

**Urban**	113	295	145	283	233	208	

	614				103		

	815				110		

	816				83		

	917				250		70

	1123+				365		

**Rural**	1324			90	88		245

	1326	85		140	100	98	113

	1330+				200		103

	1331			150	193		318

	1332			243		343	

	1341	83		100		210	168

	1342			430			

	1345			95			

	1351+				70		

All 30 volunteers were tested with DR peptides from CSP (four), SSP2/TRAP (nine), EXP1 (two), LSA1 (five) and LSA3 (two), except for volunteer 1340, who was not tested against LSA3. Six of 13 urban and nine of 17 rural volunteers had ELISpot activities that were identified as positive and are listed in this table. Each number represents the mean of quadruplicate wells in a single assay expressed as sfc/m, with medium controls subtracted. A "+" next to the volunteer identification number in the second column indicates patent parasitemia at the time the sample was taken [see Methods]. The peptides that were not recognized by any of the volunteers as well as the volunteers not having any positive responses are shown in Additional File 5.

##### Magnitude of ELISpot responses to short HLA A- and B-restricted peptides from each antigen

Only a few positive responses to short HLA A- and B-restricted peptides were identified (Table 5) and this may reflect the more limited numbers of volunteers and HLA-matched peptides that were tested. 2/8 (25%) urban volunteers (both HLA-A 02) recognized three short Class I-restricted peptide mixtures from CSP, SSP2/TRAP and LSA3. Three of nine (33%) rural volunteers (HLA A03 and HLA B53) recognized five short Class-I restricted peptides (all single peptides) from CSP, SSP2/TRAP, LSA1 and LSA3 (Table 5). Activities with short peptides ranged from 73 to 258 sfc/m. Responses of urban and rural volunteers to short peptides were similar (*p *= 0.65 Mann Whitney U test two-tailed). Responses of urban and rural volunteers to short and long peptides were also similar in both groups of volunteers (*p *= 0.58 and *p *= 0.39 for urban and rural, respectively, Mann Whitney U test two-tailed). The sequences of all positive long and short peptides are shown in Additional File 4. In total, 14/32 (43.8%) short peptides and 22/25 (88%) long peptides were active in these ELISpot assays, either as single peptides or as part of mixtures of peptides (Additional Files 4 and 5).

**Table 5:** Individual urban and rural volunteers' ELISpot activity with HLA A or HLA B-matched short Class I-restricted peptides

		CSP		SSP2/TRAP		LSA1	LSA3	
**Site**	**Vol**.	**D4, D5**	**D6**	**D12-D14**	**D15**	**D29**	**D31-D35**	**D37**

**Urban**	506	173				NT	180	

	510			73		NT		

**Rural**	1326		95		88		NT	NT

	1331							93

	1341	NT	NT	NT	NT	258		208

HLA-matches were defined for an HLA A or B allele. Two of 13 urban (506 and 510) and three of 17 rural (1326, 1331 and 1341) volunteers had positive ELISpot responses with HLA-matched short Class I-restricted peptides. Each number represents the mean of quadruplicate wells in a single assay with medium subtracted. No volunteer was infected with malaria. None of the twelve volunteers tested with EXP1 peptides were positive. NT = Not Tested:

##### Effect of parasitaemia on ELISpot activity

Six individuals in the rural population had malaria parasites when venous blood samples were drawn, and three (50%) were positive with long DR peptides (Table 4), but none were positive with class I peptides, suggesting that the frequency of ELISpot responses to class I peptides may be influenced by circulating parasites.

##### Comparison of ELISA and ELISpot activities

When ELISA (Additional Files 2 and 3) using method 1 and ELISpot data (Tables 4 and 5) were examined, almost all urban and rural volunteers with positive ELISpot activities were also positive with the same antigen in ELISA, except v506 and v816 who were negative in ELISA and positive in ELISpot (with CSP D4, D5; D64-68 respectively). This may only reflect the high frequency of positive volunteers identified using method 1. However, when the more stringent method 2 analysis of positive ELISA activities was used (Additional File 3) the lower numbers of positive assays with CSP, SSP2 and EBA175 reduced this concordance, although 10/11 positive ELISpot assays with LSA-1 remained positive in ELISA.

#### Part B

##### Volunteers and sample schedules

The objective of Part B was to collect PBMC at two time points and to perform ELISpot assays of cells from each of the two time points on three separate days. Twelve volunteers were initially enrolled, but only seven volunteers were used for this analysis as these seven completed blood draws when scheduled and sufficient blood was obtained to conduct nearly all the planned assays for one or both antigens. There were five with complete samples for both antigens, one with complete samples for CSP only (totalling six for CSP) and one with complete samples for AMA1 only (totalling six for AMA1). The sample availability is shown in Table 6.

**Table 6:** Part B assay schedule

	CSP SAMPLE 1			AMA SAMPLE 1			CSP SAMPLE 2			AMA SAMPLE 2			
Day	0	0	0	9	9	9	14	14	14	21	21	21	

Assay	1	2	3	1	2	3	1	2	3	1	2	3	TOTAL

1001				X	X	X				X	X	X	6
									
1024	X	X	X	X	X	X	X	X	X	X	X	X	12
1036	X	X	X	X		X	X	X	X	X	X	X	11
1039	X	X	X	X	X	X	X	X	X	X	X	X	12
									
1041	X	X	X				X	X	X				6
									
1054	X	X	X	X	X	X	X	X	X	X	X	X	12
1056	X	X	X	X	X	X	X	X	X	X	X	X	12

TOTAL	6	6	6	6	5	6	6	6	6	6	6	6	71

Each volunteer was sampled at different time points (for CSP day 0 and day 14; AMA1 day 9 and day 21). Frozen PBMC from each time point were tested in ELISpot assays with CSP or AMA peptide pools in three assays 1, 2 and 3 on different days as indicated. Assays used for analysis are boxed. X indicates where that volunteer was tested with CSP or AMA1 pools. The seven volunteers in the table were analysed for ELISpot reproducibility at each of the two time points (Supplementary Tables 1, 2 and 3). Totals refer to the numbers of assays used in this analysis. An additional five volunteers enrolled in the study and tested at some time points were excluded from this table and from the consistency analysis because data sets were not sufficiently complete.

PBMC were collected at days 0, 9, 14 and 21 and frozen. Assays were performed on frozen samples after all samples had been collected. Samples from day 0 and day 14 were tested in ELISpot assays with CSP peptide pools, and samples from days 9 and 21 were tested with AMA1 peptide pools. Three assays, labeled assays 1, 2 and 3, were performed on three separate days for each time-point, starting in each case with freshly thawed PBMCs. Thus with six volunteers for CSP (v1024, v1036, v1039, v1041, v1054 and v1056), 36 assays performed with 9 CSP peptide pools for a total of 324 individual assays; and with six volunteers for AMA1 (v1024, v1036, v1039, v1054, v1056 and v1001), 35 assays were performed with 12 peptide pools (v1036 was only tested in two assays at the day 9 time point due to shortage of PBMC) for a total of 420 individual assays. In addition there were 71 medium controls assayed for the seven volunteers.

##### Variability in medium controls

**Figure 3:**
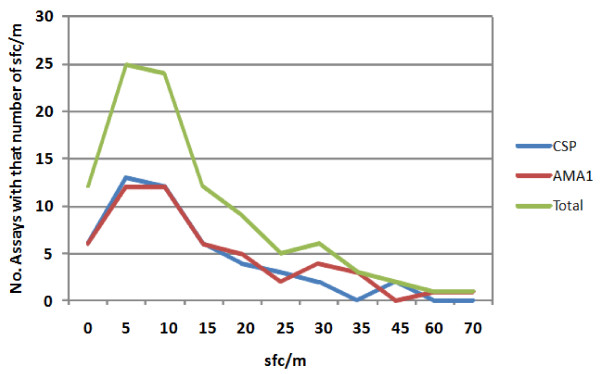
**The frequency distribution of the medium controls (sfc/m PBMC) for all the CSP and AMA1 assays**. The numbers of medium controls from each assay are distributed against the ELISpot activity (sfc/m). The distribution of medium controls from all assays using CSP or AMA1 peptide pools, and the total using both antigens, show the same distribution.

The first step was to determine the variation in ELISpot responses in control assays when PBMC's were stimulated with medium alone to establish a baseline against which positive ELISpot activities could be statistically calculated. The values of the medium only controls were examined for each of the 12 volunteers, for CSP and for AMA1, at the two time-points each with three assays (100 control assays in total, given that not all volunteers were sampled at each time point). The frequency distribution of the medium controls is shown in Figure 3. When examined by antigen, they were nearly identical (Figure 3), with most values between 0-15 sfc/m. This indicated a high degree of similarity regarding how the assays performed on different days, and therefore that assay results for different antigens or from different days could appropriately be compared.

### Reproducibility of assays

When ELISpot activities of all peptide pools were analysed, 138/324 (42.6%) of CSP assays and 236/420 (56.2%) of AMA1 assays were greater than medium controls (Table 7). The remaining peptide-pool activities were similar to medium controls, and could be considered as natural variability or "noise" of the ELISpot assay. The next step was to differentiate which activities were significantly different from medium controls and three sets of criteria were applied (See Methods).

**Table 7:** Summary of assays with positive ELISpot activities using statistical Methods 1, 2 and 3

	CSP			AMA1		
**Method**	**Assays**	**No. Positive**	Cp1*	**Assays**	**No. Positive**	Ap7and9**

**>Med**.	324	138 (42.6%)	30/138 (21.7%)	420	236 (56.2%)	53/201 (26.3%)

**1**	324	30 (9.3%)	16/30 (53.0%)	420	94 (22.4%)	40/84 (47.6%)

**2**	324	17 (5.2%)	13/17 (76.5%)	420	62 (14.8%)	34/57 (60.0%)

**3**	324	10 (3.1%)	9/10 (90%)	420	41 (9.8%)	29/36 (80.1%)

ELISpot activities >medium controls were analysed using Methods 1, 2 and 3 (see Methods) and the positive activities for each method are shown. The numbers of Cp1 are for all volunteers tested, Ap7+Ap9 excluding v1001 who responded to different peptide pools. * Proportion of positive assays that are positive by Cp1; ** Proportion of positive assays that are positive by Ap7 or Ap9 excluding v1001.

### Method 1

**Figure 4:**
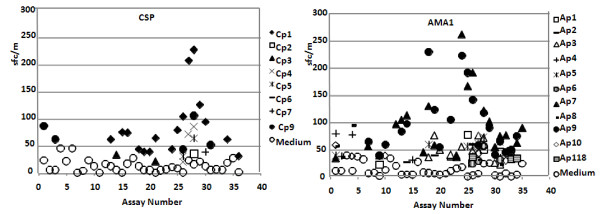
**Method 1: mean ELISpot activity of all positive assays of peptide pool stimulated PBMC compared with medium-only controls**. ELISpot activities of each set of individual volunteers are plotted against their corresponding medium only controls. Each symbol represents the mean of the triplicates and the black dotted line represents >55 sfc/m activity.

The simplest and least stringent method was to subtract the average of medium control activity from the average of antigen-stimulated activity and use a net difference of 20 sfc/m as the arbitrary cut-off [50]. The results are summarized in Table 7 and Figure 4 and details are provided in Additional File 6.

#### Positive volunteers

**Figure 5:**
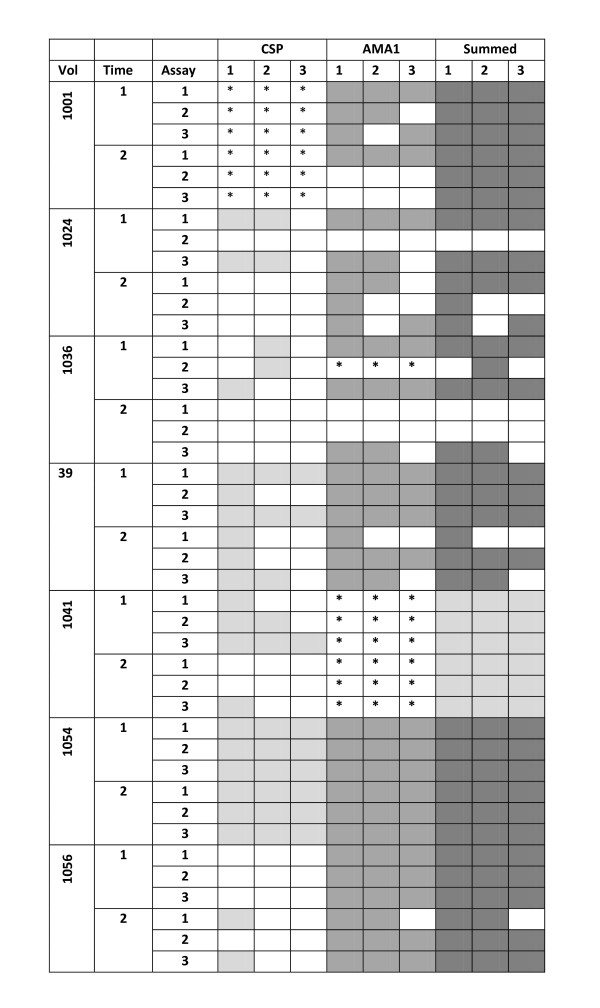
**Positive ELISpot activities with CSP or AMA1 peptide pools with Methods 1, 2 or 3**. The top three rows for each volunteer denote the three replicate assays for the first time point, and the second three rows denote the three replicate assays for the second time-point. An assay is considered positive if at least one peptide pool was positive using that method. All positive CSP and AMA1 activities are shown in light or medium gray respectively. The responses of the five volunteers (v1024, v1036, v1039, v1054 and v1056, for whom both CSP and AMA1 assays were performed) were combined and shown in dark gray. *Assay not done.

Thirty/324 (9.3%) assays with CSP and 94/420 (22.4%) assays with AMA1 pools were positive at this cut off (Table 7, details in Additional File 6). When all CSP positives were combined, v1054 was consistently positive (positive in all three assays) at both time-points, and v1039 and v1041 were consistently positive only at a single time point (Figure 5, details in Additional File 6). When positive responses to AMA1 were combined, v1039, v1054 and v1056 were consistently positive (positive in all three assays) at both time points, and v1001 and v1024 were consistently positive at one time point. v1036 was positive in both available samples at the first time-point. The remaining assays were positive at one or two of three assays at a given time-point.

#### Negative volunteers

With CSP v1036 was consistently negative (0/3 assays) at both time-points and 1024 and v1056 were consistently negative at single time-points. None were consistently negative with AMA1.

#### Positive peptide pools

**Figure 6:**
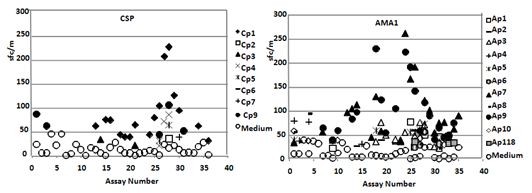
**Method 1: Linkage of ELISpot activities**. **Left panel**: ELISpot activities of Ap7 and Ap9 show moderate linkage (r^2 ^= 0,63). **Right panel**: no linkage between Ap7 and Cp1 (r^2 ^= 0.03).

When responses to individual peptide pools were examined (Table 7, details in Additional File 6), Cp1 was the most frequently positive CSP peptide pool (16/30, 53%) whereas other CSP peptide pools were less frequently positive. For AMA1, five volunteers (v1024, v1036, v1039, v1054 and v1056) recognized Ap7 and Ap9 in 40 of the total 84 positive assays (47.6%), and only one of the 30 positive assays were not positive with Ap7 and Ap9 combined. Activities to Ap7 and Ap9 (that do not overlap) appeared to be linked (R^2 ^= 0.63, Chi-square, two-tailed p = 0.79) whereas no other peptide pools were similarly linked (Figure 6). The 6^th ^volunteer (v1001) primarily recognized Ap4 and Ap8 in 3/6 assays.

### Method 2

The second method used Student's *t *test, a ratio of test wells to medium controls of greater than two, and a greater than 10 sfc/m difference over medium control and was also used in Part A.

#### Positive volunteers

This method identified 17/324 (5.2%) CSP and 62/420 (14.8%) AMA1 assays as positive (Table 7, details in Additional File 7), and these frequencies were lower than using Method 1. This method identified consistent results with CSP (3/3 assays positive) with v1054 (both time points) and with AMA1 with v1054 and v1056 (both time points positive) and v1039 (single time points) and v1036 (2 positive assays of 2 available samples) (Figure 5, details in Additional File 7).

#### Negative volunteers

v1056 was consistently negative (0/3 assays) at both time points with CSP, and v1024, v1036 and v1041 were consistently negative at one time-point. However, no volunteers were consistently negative with AMA1.

#### Positive peptide pools

For CSP, only Cp1 and Cp9 induced positive activities, and most were induced by Cp1 (13/17 and 76.5%, respectively, Figure 5, details in Additional File 7). For AMA1, Ap7 and Ap9 together induced most positive activities for 5 volunteers excluding v1001 (34/57 and 60.0%, respectively), and other AMA1 peptide pools were only positive when Ap7 and Ap9 were positive. v1001 only recognized Ap4 and Ap8.

### Method 3

This was the most stringent and used two criteria: 55 sfc/m as a minimum cut off and a 4-fold or greater difference between test wells and medium controls.

#### Positive volunteers

This method identified 10/324 (3.1%) CSP and 41/420 (9.8%) AMA1 positive assays (Table 7, details in Additional File 5), and these were lower than using Methods 1 or 2. This method gave the lowest frequency of consistent outcomes (all three sets at either time point). v1054 was positive with CSP and AMA1 with all three assays at both time points; v1039 and v1056 gave consistent positives with AMA1 in all assays at one time point, and v1036 was positive with both available samples at one time-point (Figure 5, details in Additional File 5).

#### Negative volunteers

With CSP, v1024, v1036 and v1056 were consistently negative (0/3 assays) at both time points, and v1039 and v1041 were consistently negative at one time-point. Only v1036 was consistently negative with AMA1 at one time point.

#### Positive peptide pools

For CSP, Cp1 induced 9/10 positive activities (90%) and Cp1 induced 1 positive activity. With AMA1 29/36 (80.1%) were positive with Ap7 and Ap9 combined, excluding v1001 (Table 7, details in Additional File 5). The numbers of positive assays with Ap7 and Ap9 were least affected by increasing the stringency of the analytical method used.

### Combined ELISpot responses to CSP and AMA1

**Figure 7:**
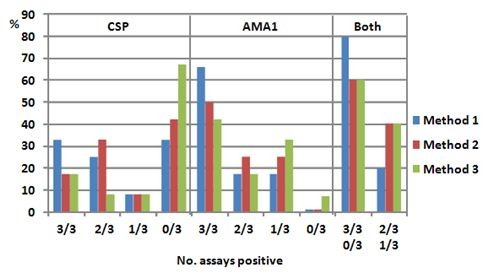
**Comparison of positive assays using Methods 1, 2 and 3**. The number of time-points with assays that were all positive (3/3), all negative (0/3) or 1/3 or 2/3 positive using Methods 1, 2 or 3 are expressed as percent of total number. For CSP and AMA1, the numbers of time-points that were 3/3 positive was highest using Method 1 and dropped with Method 2 and was lowest with Method 3. Conversely, the number of time-points that were all 3/3 negative was lowest with Method 1 and rose with Method 2 and was highest with Method 3. When CSP and AMA1 were combined, Method 1 identified more 3/3 positive or 3/3 negative (0/3 positive) time-points than Methods 2 or 3.

CSP and AMA1 responses were added together (Figure 5) for those 5 volunteers who were tested with both peptides (v1024, v1036, v1039, v1054 and v1056). Since both samples of v1036 at one time-point were consistently positive with AMA1 it was included within the 3/3 positive group to give 10 time-points. The consistent (3/3 and 0/3) and the inconsistent outcomes (2/3 and 1/3) were combined (Figure 7). Method 1 gave the greatest numbers of consistent (80%) and fewest numbers of inconsistent (20%) outcomes compared to Methods 2 and 3 (60% and 40% consistent, respectively).

### Comparison of the Two Time Points

There was only modest consistency when two time points approximately two weeks apart were compared. Each time point was classified as positive (3/3 assays positive), negative (0/3 assays positive) or indeterminate (2/3 or 1/3 assays positive), then the number of positive, negative or indeterminate results that were concordant between the two time points were calculated. For Method 1 the number of consistent assays that were positive, negative or indeterminate were 4, 1 and 0, respectively or 5/12 overall (both antigens); for Method 2: the numbers were 3, 1 and 2, respectively, or 6/12 overall; and for Method 3: the numbers were 2, 3 and 2, respectively, or 7/12 overall. Thus, overall, in only about half the cases were the results at one time-point reproduced at the second time point two weeks later.

## Discussion

Malaria vaccine development is focused on a variety of candidate antigens including those in this study. In particular, prime-boost strategies designed to elicit antibodies and especially T cell responses associated with protection are moving to immunogenicity and efficacy studies in endemic populations. A long-term goal of these studies is to establish antibody and T cell assays at sites in Southern Ghana that might be used for clinical trials[51,52]. To achieve this there were two aims. Firstly (Part A) to establish the ELISA, IFA and ELISpot assays at this study site, and secondly (Part B) to develop the ELISpot assay best suited to test potential vaccine cohorts especially where naturally-acquired activities are low and without requiring HLA typing of the volunteers. ELISpot assays have been used widely in measuring vaccine-induced T cell activities in non-endemic populations where background (pre-immunization) activities are usually low compared to those in malaria-endemic populations, and there is little information concerning the reproducibility of ELISpot assays in endemic populations.

### Antibody assays

Using a panel of recombinant proteins representing seven candidate vaccine antigens, ELISA detected relatively low antibody responses that trended higher in rural Mampong-Akwapim than urban Accra, a difference that was statistically significant only for MSP3, suggesting that the higher malaria prevalence in the rural population had only a marginal association with increased antibody responses to most antigens. IFA assays detected anti-sporozoite and anti-red blood cell antibodies with roughly equal frequency in volunteers from the two study sites, and IFA titres correlated with ELISA activities. Most adults possessed demonstrable anti-malaria antibodies as would be expected in an endemic adult population (see for example[30,53,54] where malaria transmission occurs throughout the year). When two criteria of lower and higher stringency were used to classify ELISA responses as positive or negative, all volunteers were positive to at least one antigen using either method. However, positive responses to the pre-erythrocytic antigen CSP, but not SSP2/TRAP, were rare in urban volunteers compared to rural volunteers, perhaps indicating that rural volunteers were being bitten more frequently by infected mosquitoes. The numbers of positive antigens decreased when stringency increased particularly with CSP. This analysis may suggest that the lower stringency analysis was more useful to distinguish rural and urban communities with regard to pre-erythrocytic antigens. This may be supported by the observation that positive ELISA activities to these antigens using the lower stringency analysis were more concordant with ELISpot activities (see below). The importance of defining antibody titers in naturally-acquired immunity has been underscored by recent longitudinal studies that established correlations between higher IgG levels to several antigens tested in this study and reduced risk of clinical malaria in Northern Ghana[55,56], Kenya[57] and Burkina Faso[53].

### ELISpot assays (using HLA-matched peptides)

Since T cell responses to malaria antigens are often HLA-restricted[36,37], it seemed likely that the greatest sensitivity of the ELISpot assay would be achieved using HLA-matched peptides with appropriate length for stimulating Class I or Class II responses. To define background ELISpot responses to five candidate malaria vaccine antigens, Method 2 was used to identify positive outcomes. The criteria required a statistically significant difference between replicate test (peptide stimulated) wells and medium-only control wells (Students *t *test) and at least a two-fold, 10 sfc/million difference.

The numbers of urban and rural volunteers who were positive with long DR or short HLA A or B peptides was similar, although rural volunteers recognized more antigens than urban volunteers. However, CSP responses were rare, with a higher frequency of responses to LSA1 or SSP2/TRAP, particularly to DR-restricted peptides, in both urban and rural areas. This may reflect different transmission patterns in these two sites as increased transmission in the rural site may induce higher frequency of CSP responses. The average of all positive responses was >70 sfc/m, and some responses were as high as 430 sfc/m. An earlier study showed that ELISpot responses to non-HLA-matched CSP peptides in The Gambia were very low, 23 + 11 sfc/m[58], suggesting that HLA-matched peptides and volunteers may be a more sensitive assay of ELISpot responses than using individual non-matched peptides. As in other studies[25], malaria infection appeared to suppress ELISpot responses to short peptides, suggesting that the frequency of volunteers, particularly in rural areas, with class I-restricted ELISpot responses may be underestimated.

One of the challenges associated with using this approach is determining the appropriate peptides to test. We selected peptides based on the HLA of the volunteers and the availability of HLA-matched peptides. DR-binding epitopes are relatively promiscuous and bind to more than one DR haplotype [59-62], and all volunteers were tested against 5 DR peptides regardless of HLA type, although an additional 4 DR peptides were matched to a subset of volunteers based on HLA A or B epitopes contained within them. Utilizing the peptides available, only about half of the volunteers could be tested with HLA A and B matched short peptides; thus it is difficult to know if response rates might have been significantly higher had more epitopes been synthesized and tested. In addition, matching was performed on the basis of HLA type without taking into account supertype, which could have allowed a larger number of peptides to be matched to each volunteer. Overall, this approach was cumbersome and led to a complex experimental design (Additional Files 7 &8) that likely contributed to underestimating the frequency of positive responses.

### Analysis of reproducibility (using overlapping peptides)

The second aim was to determine the reproducibility of the ELISpot assay. Due to the challenges associated with using HLA-matched peptides, an alternative approach was adopted for Part B - to test all volunteers against uniform pools of overlapping peptides without HLA testing, on the assumption that such peptides would contain all T cell epitopes that could be recognized by the volunteers and that the uniform, 15 amino acid length would elicit Class I or Class II responses. The apparent dependence of CD8+ T cell responses to plasmodial antigens on CD4+ T cells ([37,63-65] may reflect that CD4+ and CD8+ T epitopes often overlap.

Usually ELISpot assays are performed once, using replicate wells. However, it is uncertain whether a single assay accurately represents T cell activities, especially in an area where they are low. To address this, two blood samples were drawn from each volunteer approximately two weeks apart, PBMC were frozen and then tested in three assays on different days. If the ELISpot assay is reproducible then all three assays at a single time point should be either all positive or all negative.

### *Ex vivo *ELISpot assays - analysis of reproducibility

Of critical importance in determining ELISpot reproducibility are the criteria used to identify which of the relatively low, infrequent and unstable ELISpot responses induced by malaria transmission[7-9,28] should be considered positive. Unlike vaccine trials, where post-immunization responses are differentiated by comparison with pre-immunization samples, naturally-acquired responses can only be differentiated by comparison with medium-only controls. Analysis of ELISpot responses using peptide pools was complex as about half of all responses were within the range of medium-only controls. In that case, the choice of statistical analysis to determine positive activities was crucial. Such analyses identify a cut off value above which an activity is considered positive. If the cut off is low more activities are likely to be determined to be positive than using a high cut off. To explore this effect, three statistical methods of increasing stringency were applied. The least stringent method (Method 1, difference of 20 sfc/m between medium control and test sample) gave the greatest number of consistently positive (3/3) assays and the fewest where 1/3 or 2/3 were positive (Figure 7). More stringent analyses such as the Student's *t *test that has been used in vaccine trials[48] and was used in Part A, or a defined ELISpot activity that was four times and at least 55 sfc/million greater than the median of medium only controls[35], identified fewer positive assays and fewer instances when three replicate assays were consistently positive. Overall, the least stringent method yielded 80% consistent results while the other two yielded 60% consistent results, indicating that at least in this population, a simple assessment relying on a 20 sfc/million difference may be most suitable.

More volunteers were positive with AMA1 than CSP, consistent with previous studies that T cell immunity to blood stage antigens was detected in 67% of individuals[8] and less frequently with pre-erythrocytic antigens[9,28]. Although not an objective of this study, this high frequency of T cell responses to AMA1 may be associated with the effect of naturally acquired immunity on reducing severe disease[66].

Three peptide pools appeared to detect responses in most volunteers; Cp1, Ap7 and Ap9. Cp1 is known to contain HLA-A02, HLA-A24 and HLA-B44 T cell epitopes[36,64], Ap7 and Ap9 also contain previously identified proliferative epitopes[24], and Ap7 also contains a CD8+ T cell restricted epitope[31]. The Cp1 responses were consistent with those elicited by the mixture of HLA-A02-restricted D4 and D5 as D4 is contained within the peptides in Cp1. In Part A using HLA-matched peptides a total of 2/13 urban volunteers showed positive responses, whereas 3/6 volunteers in Part B showed positive responses with CSP peptide pools. Although ELISpot activities of both Part A and B with peptides or peptide pools were similar (fresh cells were used in Part A and frozen cells in Part B), peptide pools may elicit positive responses in more volunteers than HLA-matched peptides. This is not surprising as Cp1 contains at least 3 known HLA-restricted epitopes and may thus be better able to detect responses in a wider population than using a few HLA-matched peptide epitopes. Responses to Ap7 and Ap9 appeared to be linked and both pools appeared to contain immunodominant epitopes,

It remains to be demonstrated whether vaccines induce the same dominant responses in volunteers in malaria-endemic areas as have been induced in those volunteers through natural transmission. Thus, in any potential vaccine trials in Ghana, ELISpot assays may have to be further refined to distinguish vaccine-induced T cell immunity from that naturally acquired.

ELISpot responses varied significantly over even short periods of time. When the proportion of consistently positive and consistently negative assays were compared between the two time points, which were separated by just two weeks, only in about half the cases were the outcomes the same. This is consistent with the findings in other studies that T cell responses are relatively unstable over time[8,28,50]. It is not possible to quantify the degree to which these differences may have been affected by assay variation.

Recently, some of the most powerful studies have used protein arrays to measure the responses to many antigens simultaneously, identifying antigen clusters for which immunological recognition may imply clinical resistance to malaria[67,68] and this approach may be highly applicable to further studies at the sites in Ghana. The interpretation of array data faces the same questions regarding criteria for positivity as addressed in the current study.

## Conclusions

This study demonstrated that antibody assays, ELISA and IFA, could successfully measure antibodies in Ghana to malaria antigens undergoing vaccine development. Likewise, the *ex vivo *ELISpot assay was shown to reproducibly measure T cell IFN-γ activities despite responses being low and variable, but reproducibility depended partly on the statistical method used to determine positive activities. Due to ease of use, overlapping peptides appeared to be preferable to HLA typing and the use of HLA-matched peptides. For ELISA, the more stringent criteria for positivity may have better distinguished rural and urban populations, at least for pre-erythrocytic stage antigens. For ELISpot, the least stringent method tended to yield the most consistently reproducible outcomes. For both humoral and cellular assays, further work is needed to demonstrate the generalizability of these findings. Better understanding of how to conduct these assays in endemic areas is important given the requirement for their use in malaria vaccine studies at these sites.

## Competing interests

The authors declare that they have no competing interests.

## Authors' contributions

Conceived the study: MS, WOR and KAK; Designed the experiments: MS, TR, WOR, JE, DD, KAK, BDA; Served as Principal investigators: DD and MS; Served as Investigators: KAK, JO, BDA, BG, and JE; Performed Immunological assays: DD, MS, DA, HG, SA, JL, GB, RS; Provided recombinant proteins: SK; Performed statistical analyses: DB and MRH; Wrote the manuscript: WOR, MRH, MS, and TLR. All authors read and approved the final manuscript.

## Supplementary Material

Additional File 1 **Part A: Volunteers tested in IFA, ELISA and ELISpot Assay**. Thirty five volunteers met selection criteria and were used in antibody (IFA and ELISA) assays. Thirty of these volunteers were HLA-typed and used in ELISpot assays. Click here for file

Additional File 2 **Positive ELISA activities defined using Method 1**. Positive ELISA activities were defined as the mean Ghanaian volunteer OD ≥ mean control sera + 3 SD. Shaded cells show positive assays with each antigen. The numbers of positive assays per volunteer, the total number of positive assays for urban and rural populations and the mean number of positive assays/volunteer for each population are shown in the two right columns. The total numbers of urban and rural volunteers positive with each protein are shown in the bottom rows. A "+" next to the volunteer identification number in the second column indicates patent parasitemia at the time the sample was taken [see Methods]. Volunteers tested in ELISpot assays using DR-binding or HLA A and B-matched peptides shown by X and @ (Tables 3 and 4), and these indicate positive assays with each volunteer. Click here for file

Additional File 3 **Positive ELISA activities defined using Method 2**. Positive ELISA activities were defined as the mean Ghanaian volunteer OD ≥ mean control sera + 3 SD *and *at minimum titer of 100. Shaded cells show positive assays with each antigen. The numbers of positive assays per volunteer, the total number of positive assays for urban and rural populations and the mean number of positive assays/volunteer for each population are shown in the two right columns. The total numbers of urban and rural volunteers positive with each protein are shown in the bottom rows. A "+" next to the volunteer identification number in the second column indicates patent parasitemia at the time the sample was taken [see Methods]. Volunteers tested in ELISpot assays using DR-binding or HLA A and B-matched peptides shown by X and @ (Tables 3 and 4), and these indicate positive assays with each volunteer. Click here for file

Additional File 4 **Part A: Sequences of all short and long peptides tested in ELISpot assays**. Peptides were either used alone or in mixtures as indicated by horizontal divisions in the second and last columns. Each was assigned a number (last column) that was used to identify which was tested with each volunteer (Additional Table 5). Shaded cell indicate peptides or peptide mixture that elicited positive ELISpot activities in at least one volunteer. *Peptides D44 and D49 were used together. Click here for file

Additional File 5 **Urban and rural volunteers: HLA-A and HLA-B supertypes and HLA matched peptides**. Volunteers had low to medium resolution HLA typing (see Methods) and tested with HLA-matched peptides (gray cells). DR of each volunteer is not shown. Positive assays are shown in black cells. *Uncertain assignment. NA Not available. To identify the peptides for each column, refer to Additional Table 4 using the reference number provided in the second row (1-27). Click here for file

Additional File 6 **ELISpot activities to CSP or AMA1 peptide pools in replicate assays using Method 1**. ELISpot activities were determined by subtracting the medium controls from the test peptide pool, and used an arbitrary cut off of a net value of 20 sfc/m. Positive outcomes for each set are shown in light gray (CSP) and medium gray (AMA1); individual activities were combined to give a total CSP (C, light gray) or AMA1 (A, medium gray) response. V = volunteer ID; T = time-point; A = assay number. The first three rows for each volunteer are the three assays for the first time-point, while the second three rows are the assays for the second time point. Missing samples are indicated by dots. Click here for file

Additional File 7 **ELISpot activities to CSP or AMA1 peptide pools in replicate assays using Method 2**. ELISpot activities were determined using the Student's *t *test to analyze specific differences (*p *= <0.05, two tailed) between test peptide pool and medium controls, and were considered positive if the test activity was at least twice that of the medium controls and the difference was at least 10 sfc/m. Positive outcomes for each set are shown in gray; individual activities were combined to give a total CSP (C, light gray) or AMA1 (A, medium gray) response. V = volunteer ID; T = time-point; A = assay number. The first three rows for each volunteer are the three assays for the first time-point, while the second three rows are the assays for the second time point. Missing samples are indicated by dots. Click here for file

Additional File 8 **ELISpot activities to CSP or AMA1 peptide pools in replicate assays using Method 3**. ELISpot activities were positive if the median of peptide pools was >55 sfc/m and at least four times the median of the medium controls. Positive outcomes for each set are shown in gray; individual activities were combined to give a total CSP (C, light gray) or AMA1 (A, medium gray) response. V = volunteer ID; T = time-point; A = assay number. The first three rows for each volunteer are the three assays for the first time-point, while the second three rows are the assays for the second time point. Missing samples are indicated by dots. Click here for file
