# Supplementary material for: Measuring naturally acquired immune responses to candidate malaria vaccine antigens in Ghanaian adults
Source: Malar J. 2011 Jun 20;10:168. doi: 10.1186/1475-2875-10-168 (PMC3132199; doi:10.1186/1475-2875-10-168)
Supplement: Additional File 1 — Part A: Volunteers tested in IFA, ELISA and ELISpot Assay. Thirty five volunteers met selection criteria and were used in antibody (IFA and ELISA) assays. Thirty of these volunteers were HLA-typed and used in ELISpot assays. [file 1475-2875-10-168-S1.DOC]

**Additional Table 1**. Part A: Volunteers tested in IFA, ELISA and ELISpot

|  |  |  | **Assays tested** | | |
| --- | --- | --- | --- | --- | --- |
| **Site** | **Volunteer** | **Parasitemia** | **IFA** | **ELISA** | **ELISpot** |
| **Urban** | 102 |  |  |  |  |
|  | 113 |  |  |  |  |
|  | 205 |  |  |  |  |
|  | 506 |  |  |  |  |
|  | 507 |  |  |  |  |
|  | 508 |  |  |  |  |
|  | 509 |  |  |  |  |
|  | 510 |  |  |  |  |
|  | 614 |  |  |  |  |
|  | 815 |  |  |  |  |
|  | 816 |  |  |  |  |
|  | 917 |  |  |  |  |
|  | 1119 |  |  |  |  |
|  | 1123 | **+** |  |  |  |
|  | **Sub tot.** | **1** | **14** | **14** | **13** |
| **Rural** | 1324 |  |  |  |  |
|  | 1325 |  |  |  |  |
|  | 1326 |  |  |  |  |
|  | 1327 |  |  |  |  |
|  | 1330 | **+** |  |  |  |
|  | 1331 |  |  |  |  |
|  | 1332 |  |  |  |  |
|  | 1334 |  |  |  |  |
|  | 1336 |  |  |  |  |
|  | 1337 |  |  |  |  |
|  | 1339 | **+** |  |  |  |
|  | 1340 | **+** |  |  |  |
|  | 1341 |  |  |  |  |
|  | 1342 |  |  |  |  |
|  | 1343 | **+** |  |  |  |
|  | 1344 |  |  |  |  |
|  | 1345 |  |  |  |  |
|  | 1346 |  |  |  |  |
|  | 1349 |  |  |  |  |
|  | 1350 |  |  |  |  |
|  | 1351 | **+** |  |  |  |
|  | **Sub tot.** | **5** | **21** | **21** | **17** |
| **Total** |  | **6** | **35** | **35** | **30** |

Thirty five volunteers met selection criteria and were used in antibody (IFA and ELISA) assays. Thirty of these volunteers were HLA-typed and used in ELISpot assays.
