# Supplementary material for: Measuring naturally acquired immune responses to candidate malaria vaccine antigens in Ghanaian adults
Source: Malar J. 2011 Jun 20;10:168. doi: 10.1186/1475-2875-10-168 (PMC3132199; doi:10.1186/1475-2875-10-168)
Supplement: Additional File 2 — Positive ELISA activities defined using Method 1. Positive ELISA activities were defined as the mean Ghanaian volunteer OD ≥ mean control sera + 3 SD. Shaded cells show positive assays with each antigen. The numbers of positive assays per volunteer, the total number of positive assays for urban and rural populations and the mean number of positive assays/volunteer for each population are shown in the two right columns. The total numbers of urban and rural volunteers positive with each protein are shown in the bottom rows. A "+" next to the volunteer identification number in the second column indicates patent parasitemia at the time the sample was taken [see Methods]. Volunteers tested in ELISpot assays using DR-binding or HLA A and B-matched peptides shown by X and @ (Tables 3 and 4), and these indicate positive assays with each volunteer. [file 1475-2875-10-168-S2.DOC]

**Additional Table 2: Positive ELISA activities defined using Method 1**

|  |  |  | **Antigen** | | | | | | |  |  |
| --- | --- | --- | --- | --- | --- | --- | --- | --- | --- | --- | --- |
| Site | **Vol** | **ELIspot** | **CSP** | **SSP2** | **EXP1** | **LSA1** | **MSP1** | **MSP3** | **EBA** | **Total** | **No./vol** |
| Urban | 102 | X |  |  |  |  |  |  |  | 6 |  |
|  | 113 | X@ | X | X |  | x |  |  |  | 7 |  |
|  | 205 | X@ |  |  |  |  |  |  |  | 4 |  |
|  | 506 | X@ | @ |  |  |  |  |  |  | 6 |  |
|  | 507 | X@ |  |  |  |  |  |  |  | 3 |  |
|  | 508 | X@ |  |  |  |  |  |  |  | 2 |  |
|  | 509 | X@ |  |  |  |  |  |  |  | 5 |  |
|  | 510 | X@ |  | @ |  |  |  |  |  | 5 |  |
|  | 614 | X |  |  |  | X |  |  |  | 5 |  |
|  | 815 | X |  |  |  | X |  |  |  | 6 |  |
|  | 816 | X |  |  |  | X |  |  |  | 2 |  |
|  | 917 | X |  |  | X | X |  |  |  | 6 |  |
|  | 1119 | X |  |  |  |  |  |  |  | 6 |  |
|  | 1123 + | X |  |  |  | X |  |  |  | 6 |  |
|  | **Sub tot** |  | **2** | **10** | **11** | **12** | **11** | **14** | **8** | **68** | **4.9** |
| Rural | 1324 | X@ |  | X | X | X |  |  |  | 7 |  |
|  | 1325 | X |  |  |  |  |  |  |  | 3 |  |
|  | 1326 | X@ | X@ | X@ |  | X |  |  |  | 6 |  |
|  | 1327 | X |  |  |  |  |  |  |  | 7 |  |
|  | 1330 + | X@ |  |  | X | X |  |  |  | 6 |  |
|  | 1331 | X@ |  | X | X | X |  |  |  | 7 |  |
|  | 1332 | X |  | X |  |  |  |  |  | 7 |  |
|  | 1334 | X |  |  |  |  |  |  |  | 7 |  |
|  | 1336 | X@ |  |  |  |  |  |  |  | 7 |  |
|  | 1337 | X |  |  |  |  |  |  |  | 6 |  |
|  | 1339 + | X@ |  |  |  |  |  |  |  | 7 |  |
|  | 1340 + | X |  |  |  |  |  |  |  | 6 |  |
|  | 1341 | X |  | X |  | @ |  |  |  | 5 |  |
|  | 1342 | X |  | X |  |  |  |  | X | 6 |  |
|  | 1343 + | X |  |  |  |  |  |  |  | 3 |  |
|  | 1344 | X |  |  |  |  |  |  |  | 7 |  |
|  | 1345 | X |  | X |  |  |  |  |  | 2 |  |
|  | 1346 | X@ |  |  |  |  |  |  |  | 6 |  |
|  | 1349 | X@ |  |  |  |  |  |  |  | 6 |  |
|  | 1350 | X |  |  |  |  |  |  |  | 6 |  |
|  | 1351 + | X@ |  |  |  | X |  |  |  | 7 |  |
|  | **Sub tot** |  | **11** | **21** | **19** | **20** | **17** | **20** | **16** | **124** | **5.9** |
| **Total** |  |  | **13** | **31** | **30** | **32** | **28** | **34** | **24** | **192** | **5.5** |

Positive ELISA activities were defined as the mean Ghanaian volunteer OD > mean control sera + 3 SD. Shaded cells show positive assays with each antigen. The numbers of positive assays per volunteer, the total number of positive assays for urban and rural volunteers and the number of positive asssays/urban pr rural volunteers are shown in the two right panels. The total numbers of urban and rural volunteers positive with each protein are shown as subtotal (sub tot) and total for both in the bottom two rows. + indicates volunteers who were parasitemic at the time the sample was taken. Volunteers tested in ELISpot assays using DR-binding or HLA A and B-matched peptides shown by X and @ (Tables 3 and 4), and these indicate positive assays with each volunteer.
