# Supplementary material for: Measuring naturally acquired immune responses to candidate malaria vaccine antigens in Ghanaian adults
Source: Malar J. 2011 Jun 20;10:168. doi: 10.1186/1475-2875-10-168 (PMC3132199; doi:10.1186/1475-2875-10-168)
Supplement: Additional File 4 — Part A: Sequences of all short and long peptides tested in ELISpot assays. Peptides were either used alone or in mixtures as indicated by horizontal divisions in the second and last columns. Each was assigned a number (last column) that was used to identify which was tested with each volunteer (Additional Table 5). Shaded cell indicate peptides or peptide mixture that elicited positive ELISpot activities in at least one volunteer. *Peptides D44 and D49 were used together. [file 1475-2875-10-168-S4.DOC]

**Additional Table 4. Part A: Sequences of all short and long peptides tested in ELISpot assays**

| **Short Peptides** | | | | | **No.** |
| --- | --- | --- | --- | --- | --- |
| **Antigen** | **Peptide** | **HLA** | **Sequence** | **Amino Acids** |  |
| **CSP** | D1 | A01 | EPSDKHIKEY | 310-319 | 1 |
|  | D2  D3 | A02  A02 | GLIMVLSFL | 386-394 | 2 |
|  |  |  | ILSVSSFLFV | 7-16 |  |
|  | D4  D5 | A02 | MMRKLAILSV | 1-10 | 3 |
|  |  |  | YLNKIQNSL | 319-327 |  |
|  | D6 | A03 | VTCGNGIQVR | 336-345 | 4 |
|  | D7 | B07 | MPNDPNRNV | 285-293 | 5 |
|  | D9 | B35 | KPKDELDY | 353-360 | 6 |
| **SSP2/TRAP** | D11 | A02 | FLIFFDLFLV | 14-23 | 7 |
|  | D12  D13  D14 | A2.1 | HLGNVKYLV | 3-11 | 8 |
|  |  |  | GIAGGLALL | 515-523 |  |
|  |  |  | LLMDCSGSI | 51-59 |  |
|  | D15 | A03 | LLACAGLLAYK | 522-531 | 9 |
|  | D16 | B07 | TPYAGEPAP | 539-547 | 10 |
| **EXP1** | D18  D19  D20  D21 | A02 | VLAGLLGNV | 80-88 | 11 |
|  |  |  | KILSVFFLA | 2-10 |  |
|  |  |  | GLLGNVSTV | 83-91 |  |
|  |  |  | VLLGGVGLVL | 91-100 |  |
|  | D22 | A03 | ALFFIIFNK | 10-18 | 12 |
| **LSA1** | D23  D24  D25  D26 | A03 | QTNFKSLLR | 94-102 | 13 |
|  |  |  | GVSENIFLK | 105-113 |  |
|  |  |  | HVLSHNSYEK | 59-68 |  |
|  |  |  | FILVNKKIFH | 11-20 |  |
|  | D28 | B035 | KPNDKSLY | 1850-1857 | 14 |
|  | D29 | B53 | KPIVQYDNF | 1786-1794 | 15 |
| **LSA3** | D31  D32  D33  D34  D35 | A2.1 | DLLEEGNTL | 113-121 | 16 |
|  |  |  | KLEELHENV | 664-672 |  |
|  |  |  | GLLNKLENI | 831-839 |  |
|  |  |  | EIDITSKL | 1005-1013 |  |
|  |  |  | HIISGDAH | 1181-1189 |  |
|  | D37 | B53 | APFISAVAA | 1429-1437 | 17 |
|  | D38 | B07 | EPKDEIVEV | 1295-1303 | 18 |
| **Influenza A**  **Matrix Protein1** | 02 | A02 | GILGFVFTL | 58-66 |  |
| **Influenza A Nucleo-protein** | 03 | A03 | ILRGSVAHK | 265-273 |  |
| **Long Peptides** | | | | |  |
| **CSP** | D10 | DR | KPKDELDYANDIEKKICKKMEKCS | 353-375 | 19 |
|  | D44* |  | LEMNYYGKQENWYSLKKNSR | 51-70 | 20 |
|  | D45 |  | QGHNMPNDPNRNVDENANAN | 281-300 | 21 |
|  | D46  D47 |  | EYLNKIQNSLSTEWSPCSVT | 318-337 | 22 |
|  |  |  | PSDKHIKEYLNKIQNSLSTE | 311-330 |  |
|  | D48 |  | IKPGSANKPKDELDYANDIE | 346-365 | 23 |
|  | D49* |  | DIEKKICKMEKCSSVFNVVNS | 363-383 | 20 |
| **SSP2/TRAP** | D51  D52  D53  D54  D55  D56  D57  D58  D59 |  | RHNWVNHAVPLAMKLI | 61-76 | 24 |
|  |  |  | VKNVIGPFMKAVCVE | 223-237 |  |
|  |  |  | GLAYKFVVPGAATPY | 527-541 |  |
|  |  |  | KYKIAGGIAGGLALL | 509-523 |  |
|  |  |  | VFLIFFDLFLVNGRDVQNNI | 13-22 |  |
|  |  |  | YANIFSNNAKEIIRLHSDAS | 89-108 |  |
|  |  |  | TNLPYGRTNLTDALL | 124-138 |  |
|  |  |  | IRLHSDASKNKEKALIIIKS | 101-120 |  |
|  |  |  | TEEHEKPDNNKKKAGSDNKN | 491-510 |  |
| **Exp1** | D60  D61 |  | AGLLGNVSTVLLGGV | 82-96 | 25 |
|  |  |  | KSKYKLATSVLAGLL | 71-85 |  |
| **LSA1** | D64  D65  D66  D67  D68 |  | LTMSNVKNVQTNFKSLLRNLGVS | 84-107 | 26 |
|  |  |  | NENLDDLDEGIEKSSEELSEEKI | 1813-1835 |  |
|  |  |  | DNEILQIVKELSEKITKYFMKL | 1888-1909 |  |
|  |  |  | ERRAKEKLQEQQRDLEQRKADTKK | 1613-1636 |  |
|  |  |  | DTKKNLERKKEHGDILAEDLYGRLEIP | 1633-1659 |  |
| **LSA3** | D70  D72 |  | LLSNIEEPKENIIDNLLNNI | 142-161 | 27 |
|  |  |  | LEESQVNDDIFNSLVKSVQQEQQHNV | 200-225 |  |

Peptides were either used alone or in mixtures as indicated in the text. Each was assigned a number that was used to identify which was tested with each volunteer (Additional Table 2). Shaded cell indicate peptides or peptide mixture that elicited positive ELISpot activities (Tables 2 and 3). *Peptides D44 and D49 were used together.
