# Supplementary material for: Measuring naturally acquired immune responses to candidate malaria vaccine antigens in Ghanaian adults
Source: Malar J. 2011 Jun 20;10:168. doi: 10.1186/1475-2875-10-168 (PMC3132199; doi:10.1186/1475-2875-10-168)
Supplement: Additional File 5 — Urban and rural volunteers: HLA-A and HLA-B supertypes and HLA matched peptides. Volunteers had low to medium resolution HLA typing (see Methods) and tested with HLA-matched peptides (gray cells). DR of each volunteer is not shown. Positive assays are shown in black cells. *Uncertain assignment. NA Not available. To identify the peptides for each column, refer to Additional Table 4 using the reference number provided in the second row (1-27). [file 1475-2875-10-168-S5.DOC]

**Additional Table 5. Urban and rural volunteers: HLA-A and HLA-B supertypes** and HLA matched peptides

|  | | | **Peptide or Peptide Mixture** | | | | | | | | | | | | | | | | | | | | | | | | | | |
| --- | --- | --- | --- | --- | --- | --- | --- | --- | --- | --- | --- | --- | --- | --- | --- | --- | --- | --- | --- | --- | --- | --- | --- | --- | --- | --- | --- | --- | --- |
| **Vol** | **HLA-A** | **HLA-B** | **1** | **2** | **3** | **4** | **5** | **6** | **7** | **8** | **9** | **10** | **11** | **12** | **13** | **14** | **15** | **16** | **17** | **18** | **19** | **20** | **21** | **22** | **23** | **24** | **25** | **26** | **27** |
|  |  |  | A01 | A02 | A02 | A03 | B07 | B35 | A02 | A2.1 | A03 | B07 | A02 | A03 | A03 | B35 | B53 | A2.1 | B53 | B53 | DR | DR | DR | DR | DR | DR | DR | DR | DR |
| 113 | --/02 | 45/53 |  |  |  |  |  |  |  |  |  |  |  |  |  |  |  |  |  |  |  |  |  |  |  |  |  |  |  |
| 205 | 23/33 | 35/44 |  |  |  |  |  |  |  |  |  |  |  |  |  |  |  |  |  |  |  |  |  |  |  |  |  |  |  |
| 506 | 02/33 | 57/67 |  |  |  |  |  |  |  |  |  |  |  |  |  |  |  |  |  |  |  |  |  |  |  |  |  |  |  |
| 507 | 01/02 | 39/72 |  |  |  |  |  |  |  |  |  |  |  |  |  |  |  |  |  |  |  |  |  |  |  |  |  |  |  |
| 508 | 02/23 | 45/65 |  |  |  |  |  |  |  |  |  |  |  |  |  |  |  |  |  |  |  |  |  |  |  |  |  |  |  |
| 509 | 02/68 | 53/77 |  |  |  |  |  |  |  |  |  |  |  |  |  |  |  |  |  |  |  |  |  |  |  |  |  |  |  |
| 510 | 2.2/34 | 44/57 |  |  |  |  |  |  |  |  |  |  |  |  |  |  |  |  |  |  |  |  |  |  |  |  |  |  |  |
| 614 | 30/34 | 70/27 |  |  |  |  |  |  |  |  |  |  |  |  |  |  |  |  |  |  |  |  |  |  |  |  |  |  |  |
| 815 | 30/68 | 08/42 |  |  |  |  |  |  |  |  |  |  |  |  |  |  |  |  |  |  |  |  |  |  |  |  |  |  |  |
| 816 | 23/74 | 44/50 |  |  |  |  |  |  |  |  |  |  |  |  |  |  |  |  |  |  |  |  |  |  |  |  |  |  |  |
| 917 | 30/74 | 37/49 |  |  |  |  |  |  |  |  |  |  |  |  |  |  |  |  |  |  |  |  |  |  |  |  |  |  |  |
| 1119 | 33/68 | 44/45 |  |  |  |  |  |  |  |  |  |  |  |  |  |  |  |  |  |  |  |  |  |  |  |  |  |  |  |
| 1123 | 02/33 | 35/37 |  |  |  |  |  |  |  |  |  |  |  |  |  |  |  |  |  |  |  |  |  |  |  |  |  |  |  |
| 1324 | 33/68 | 44/53 |  |  |  |  |  |  |  |  |  |  |  |  |  |  |  |  |  |  |  |  |  |  |  |  |  |  |  |
| 1326 | 03/66 | 35/42 |  |  |  |  |  |  |  |  |  |  |  |  |  |  |  |  |  |  |  |  |  |  |  |  |  |  |  |
| 1330 | 01/01 | NA |  |  |  |  |  |  |  |  |  |  |  |  |  |  |  |  |  |  |  |  |  |  |  |  |  |  |  |
| 1331 | 24/30 | 07/57* |  |  |  |  |  |  |  |  |  |  |  |  |  |  |  |  |  |  |  |  |  |  |  |  |  |  |  |
| 1332 | NA | NA |  |  |  |  |  |  |  |  |  |  |  |  |  |  |  |  |  |  |  |  |  |  |  |  |  |  |  |
| 1336 | 03/29 | --/44 |  |  |  |  |  |  |  |  |  |  |  |  |  |  |  |  |  |  |  |  |  |  |  |  |  |  |  |
| 1337 | 30/74 | 18/45 |  |  |  |  |  |  |  |  |  |  |  |  |  |  |  |  |  |  |  |  |  |  |  |  |  |  |  |
| 1339 | 03/68 | 53/57 |  |  |  |  |  |  |  |  |  |  |  |  |  |  |  |  |  |  |  |  |  |  |  |  |  |  |  |
| 1340 | NA | NA |  |  |  |  |  |  |  |  |  |  |  |  |  |  |  |  |  |  |  |  |  |  |  |  |  |  |  |
| 1341 | 23/68 | 53/68 |  |  |  |  |  |  |  |  |  |  |  |  |  |  |  |  |  |  |  |  |  |  |  |  |  |  |  |
| 1342 | 30/74 | 42/-- |  |  |  |  |  |  |  |  |  |  |  |  |  |  |  |  |  |  |  |  |  |  |  |  |  |  |  |
| 1343 | 24/30 | --/58 |  |  |  |  |  |  |  |  |  |  |  |  |  |  |  |  |  |  |  |  |  |  |  |  |  |  |  |
| 1345 | 31/33 | 53/72 |  |  |  |  |  |  |  |  |  |  |  |  |  |  |  |  |  |  |  |  |  |  |  |  |  |  |  |
| 1346 | 03/68 | NA |  |  |  |  |  |  |  |  |  |  |  |  |  |  |  |  |  |  |  |  |  |  |  |  |  |  |  |
| 1349 | 02/30 | 07/35 |  |  |  |  |  |  |  |  |  |  |  |  |  |  |  |  |  |  |  |  |  |  |  |  |  |  |  |
| 1350 | 30/66 | 42/72 |  |  |  |  |  |  |  |  |  |  |  |  |  |  |  |  |  |  |  |  |  |  |  |  |  |  |  |
| 1351 | 30/36 | 18/35 |  |  |  |  |  |  |  |  |  |  |  |  |  |  |  |  |  |  |  |  |  |  |  |  |  |  |  |

Volunteers had low to medium resolution HLA typing (see Methods) and tested with HLA-matched peptides (gray cells). DR of each volunteer is not shown. Positive assays are shown in black cells. *Uncertain assignment. NA: Not available.
