# Supplementary material for: Measuring naturally acquired immune responses to candidate malaria vaccine antigens in Ghanaian adults
Source: Malar J. 2011 Jun 20;10:168. doi: 10.1186/1475-2875-10-168 (PMC3132199; doi:10.1186/1475-2875-10-168)
Supplement: Additional File 6 — ELISpot activities to CSP or AMA1 peptide pools in replicate assays using Method 1. ELISpot activities were determined by subtracting the medium controls from the test peptide pool, and used an arbitrary cut off of a net value of 20 sfc/m. Positive outcomes for each set are shown in light gray (CSP) and medium gray (AMA1); individual activities were combined to give a total CSP (C, light gray) or AMA1 (A, medium gray) response. V = volunteer ID; T = time-point; A = assay number. The first three rows for each volunteer are the three assays for the first time-point, while the second three rows are the assays for the second time point. Missing samples are indicated by dots. [file 1475-2875-10-168-S6.DOC]

**Additional Table 6: ELISpot activities to CSP or AMA1 peptide pools in replicate assays using Method 1**

|  |  |  | **CSP Peptide Pool** | | | | | | | | | **AMA1 Peptide Pool** | | | | | | | | | | | |  |  |
| --- | --- | --- | --- | --- | --- | --- | --- | --- | --- | --- | --- | --- | --- | --- | --- | --- | --- | --- | --- | --- | --- | --- | --- | --- | --- |
| V | **T** | **A** | **1** | **2** | **3** | **4** | **5** | **6** | **7** | **8** | **9** | **1** | **2** | **3** | **4** | **5** | **6** | **7** | **8** | **9** | **10** | **11** | **12** | **C** | A |
| **1001** | 1 | 1 | * | * | * | ***** | ***** | ***** | ***** | ***** | ***** |  |  |  |  |  |  |  |  |  |  |  |  | ***** |  |
|  |  | 2 | * | * | * | ***** | ***** | ***** | ***** | ***** | ***** |  |  |  |  |  |  |  |  |  |  |  |  | ***** |  |
|  |  | 3 | * | * | * | ***** | ***** | ***** | ***** | ***** | ***** |  |  |  |  |  |  |  |  |  |  |  |  | ***** |  |
|  | 2 | 1 | * | * | * | ***** | ***** | ***** | ***** | ***** | ***** |  |  |  |  |  |  |  |  |  |  |  |  | ***** |  |
|  |  | 2 | * | * | * | ***** | ***** | ***** | ***** | ***** | ***** |  |  |  |  |  |  |  |  |  |  |  |  | ***** |  |
|  |  | 3 | * | * | * | ***** | ***** | ***** | ***** | ***** | ***** |  |  |  |  |  |  |  |  |  |  |  |  | ***** |  |
| **1024** | 1 | 1 |  |  |  |  |  |  |  |  |  |  |  |  |  |  |  |  |  |  |  |  |  |  |  |
|  |  | 2 |  |  |  |  |  |  |  |  |  |  |  |  |  |  |  |  |  |  |  |  |  |  |  |
|  |  | 3 |  |  |  |  |  |  |  |  |  |  |  |  |  |  |  |  |  |  |  |  |  |  |  |
|  | 2 | 1 |  |  |  |  |  |  |  |  |  |  |  |  |  |  |  |  |  |  |  |  |  |  |  |
|  |  | 2 |  |  |  |  |  |  |  |  |  |  |  |  |  |  |  |  |  |  |  |  |  |  |  |
|  |  | 3 |  |  |  |  |  |  |  |  |  |  |  |  |  |  |  |  |  |  |  |  |  |  |  |
| **1036** | 1 | 1 |  |  |  |  |  |  |  |  |  |  |  |  |  |  |  |  |  |  |  |  |  |  |  |
|  |  | 2 |  |  |  |  |  |  |  |  |  | ***** | ***** | ***** | ***** | ***** | ***** | ***** | ***** | ***** | ***** | ***** | ***** |  | * |
|  |  | 3 |  |  |  |  |  |  |  |  |  |  |  |  |  |  |  |  |  |  |  |  |  |  |  |
|  | 2 | 1 |  |  |  |  |  |  |  |  |  |  |  |  |  |  |  |  |  |  |  |  |  |  |  |
|  |  | 2 |  |  |  |  |  |  |  |  |  |  |  |  |  |  |  |  |  |  |  |  |  |  |  |
|  |  | 3 |  |  |  |  |  |  |  |  |  |  |  |  |  |  |  |  |  |  |  |  |  |  |  |
| **1039** | 1 | 1 |  |  |  |  |  |  |  |  |  |  |  |  |  |  |  |  |  |  |  |  |  |  |  |
|  |  | 2 |  |  |  |  |  |  |  |  |  |  |  |  |  |  |  |  |  |  |  |  |  |  |  |
|  |  | 3 |  |  |  |  |  |  |  |  |  |  |  |  |  |  |  |  |  |  |  |  |  |  |  |
|  | 2 | 1 |  |  |  |  |  |  |  |  |  |  |  |  |  |  |  |  |  |  |  |  |  |  |  |
|  |  | 2 |  |  |  |  |  |  |  |  |  |  |  |  |  |  |  |  |  |  |  |  |  |  |  |
|  |  | 3 |  |  |  |  |  |  |  |  |  |  |  |  |  |  |  |  |  |  |  |  |  |  |  |
| **1041** | 1 | 1 |  |  |  |  |  |  |  |  |  | ***** | ***** | ***** | ***** | ***** | ***** | ***** | ***** | ***** | ***** | ***** | ***** |  | * |
|  |  | 2 |  |  |  |  |  |  |  |  |  | ***** | ***** | ***** | ***** | ***** | ***** | ***** | ***** | ***** | ***** | ***** | ***** |  | * |
|  |  | 3 |  |  |  |  |  |  |  |  |  | ***** | ***** | ***** | ***** | ***** | ***** | ***** | ***** | ***** | ***** | ***** | ***** |  | * |
|  | 2 | 1 |  |  |  |  |  |  |  |  |  | ***** | ***** | ***** | ***** | ***** | ***** | ***** | ***** | ***** | ***** | ***** | ***** |  | * |
|  |  | 2 |  |  |  |  |  |  |  |  |  | ***** | ***** | ***** | ***** | ***** | ***** | ***** | ***** | ***** | ***** | ***** | ***** |  | * |
|  |  | 3 |  |  |  |  |  |  |  |  |  | ***** | ***** | ***** | ***** | ***** | ***** | ***** | ***** | ***** | ***** | ***** | ***** |  | * |
| **1054** | 1 | 1 |  |  |  |  |  |  |  |  |  |  |  |  |  |  |  |  |  |  |  |  |  |  |  |
|  |  | 2 |  |  |  |  |  |  |  |  |  |  |  |  |  |  |  |  |  |  |  |  |  |  |  |
|  |  | 3 |  |  |  |  |  |  |  |  |  |  |  |  |  |  |  |  |  |  |  |  |  |  |  |
|  | 2 | 1 |  |  |  |  |  |  |  |  |  |  |  |  |  |  |  |  |  |  |  |  |  |  |  |
|  |  | 2 |  |  |  |  |  |  |  |  |  |  |  |  |  |  |  |  |  |  |  |  |  |  |  |
|  |  | 3 |  |  |  |  |  |  |  |  |  |  |  |  |  |  |  |  |  |  |  |  |  |  |  |
| **1056** | 1 | 1 |  |  |  |  |  |  |  |  |  |  |  |  |  |  |  |  |  |  |  |  |  |  |  |
|  |  | 2 |  |  |  |  |  |  |  |  |  |  |  |  |  |  |  |  |  |  |  |  |  |  |  |
|  |  | 3 |  |  |  |  |  |  |  |  |  |  |  |  |  |  |  |  |  |  |  |  |  |  |  |
|  | 2 | 1 |  |  |  |  |  |  |  |  |  |  |  |  |  |  |  |  |  |  |  |  |  |  |  |
|  |  | 2 |  |  |  |  |  |  |  |  |  |  |  |  |  |  |  |  |  |  |  |  |  |  |  |
|  |  | 3 |  |  |  |  |  |  |  |  |  |  |  |  |  |  |  |  |  |  |  |  |  |  |  |

ELISpot activities were determined by subtracting the medium controls from the test peptide pool, and used an arbitrary cut off of a net value of 20 sfc/m. Positive outcomes for each set are shown in gray; individual activities were combined to give a total CSP (C, light gray) or AMA1 (A, medium gray) response. V=volunteer ID; T=time-point; A=assay number. The first three rows for each volunteer are the three assays for the first time-point, while the second three rows are the assays for the second time point. Missing samples are indicated by dots.
