# Supplementary material for: Measuring naturally acquired immune responses to candidate malaria vaccine antigens in Ghanaian adults
Source: Malar J. 2011 Jun 20;10:168. doi: 10.1186/1475-2875-10-168 (PMC3132199; doi:10.1186/1475-2875-10-168)
Supplement: Additional File 8 — ELISpot activities to CSP or AMA1 peptide pools in replicate assays using Method 3. ELISpot activities were positive if the median of peptide pools was >55 sfc/m and at least four times the median of the medium controls. Positive outcomes for each set are shown in gray; individual activities were combined to give a total CSP (C, light gray) or AMA1 (A, medium gray) response. V = volunteer ID; T = time-point; A = assay number. The first three rows for each volunteer are the three assays for the first time-point, while the second three rows are the assays for the second time point. Missing samples are indicated by dots. [file 1475-2875-10-168-S8.DOC]

**Additional Table 8: ELISpot activities to CSP or AMA1 peptide pools in replicate assays using Method 3**

|  |  |  | **CSP** | | | | | | | | | **AMA1** | | | | | | | | | | | |  |  |
| --- | --- | --- | --- | --- | --- | --- | --- | --- | --- | --- | --- | --- | --- | --- | --- | --- | --- | --- | --- | --- | --- | --- | --- | --- | --- |
| V* | **T** | **A** | **1** | **2** | **3** | **4** | **5** | **6** | **7** | **8** | **9** | **1** | **2** | **3** | **4** | **5** | **6** | **7** | **8** | **9** | **10** | **11** | **12** | **C** | **A** |
| **1001** | 1 | 1 | * | * | * | ***** | ***** | ***** | ***** | ***** | ***** |  |  |  |  |  |  |  |  |  |  |  |  | ***** |  |
|  |  | 2 | * | * | * | ***** | ***** | ***** | ***** | ***** | ***** |  |  |  |  |  |  |  |  |  |  |  |  | ***** |  |
|  |  | 3 | * | * | * | ***** | ***** | ***** | ***** | ***** | ***** |  |  |  |  |  |  |  |  |  |  |  |  | ***** |  |
|  | 2 | 1 | * | * | * | ***** | ***** | ***** | ***** | ***** | ***** |  |  |  |  |  |  |  |  |  |  |  |  | ***** |  |
|  |  | 2 | * | * | * | ***** | ***** | ***** | ***** | ***** | ***** |  |  |  |  |  |  |  |  |  |  |  |  | ***** |  |
|  |  | 3 | * | * | * | ***** | ***** | ***** | ***** | ***** | ***** |  |  |  |  |  |  |  |  |  |  |  |  | ***** |  |
| **1024** | 1 | 1 |  |  |  |  |  |  |  |  |  |  |  |  |  |  |  |  |  |  |  |  |  |  |  |
|  |  | 2 |  |  |  |  |  |  |  |  |  |  |  |  |  |  |  |  |  |  |  |  |  |  |  |
|  |  | 3 |  |  |  |  |  |  |  |  |  |  |  |  |  |  |  |  |  |  |  |  |  |  |  |
|  | 2 | 1 |  |  |  |  |  |  |  |  |  |  |  |  |  |  |  |  |  |  |  |  |  |  |  |
|  |  | 2 |  |  |  |  |  |  |  |  |  |  |  |  |  |  |  |  |  |  |  |  |  |  |  |
|  |  | 3 |  |  |  |  |  |  |  |  |  |  |  |  |  |  |  |  |  |  |  |  |  |  |  |
| **1036** | 1 | 1 |  |  |  |  |  |  |  |  |  |  |  |  |  |  |  |  |  |  |  |  |  |  |  |
|  |  | 2 |  |  |  |  |  |  |  |  |  | ***** | ***** | ***** | ***** | ***** | ***** | ***** | ***** | ***** | ***** | ***** | ***** |  | * |
|  |  | 3 |  |  |  |  |  |  |  |  |  |  |  |  |  |  |  |  |  |  |  |  |  |  |  |
|  | 2 | 1 |  |  |  |  |  |  |  |  |  |  |  |  |  |  |  |  |  |  |  |  |  |  |  |
|  |  | 2 |  |  |  |  |  |  |  |  |  |  |  |  |  |  |  |  |  |  |  |  |  |  |  |
|  |  | 3 |  |  |  |  |  |  |  |  |  |  |  |  |  |  |  |  |  |  |  |  |  |  |  |
| **1039** | 1 | 1 |  |  |  |  |  |  |  |  |  |  |  |  |  |  |  |  |  |  |  |  |  |  |  |
|  |  | 2 |  |  |  |  |  |  |  |  |  |  |  |  |  |  |  |  |  |  |  |  |  |  |  |
|  |  | 3 |  |  |  |  |  |  |  |  |  |  |  |  |  |  |  |  |  |  |  |  |  |  |  |
|  | 2 | 1 |  |  |  |  |  |  |  |  |  |  |  |  |  |  |  |  |  |  |  |  |  |  |  |
|  |  | 2 |  |  |  |  |  |  |  |  |  |  |  |  |  |  |  |  |  |  |  |  |  |  |  |
|  |  | 3 |  |  |  |  |  |  |  |  |  |  |  |  |  |  |  |  |  |  |  |  |  |  |  |
| **1041** | 1 | 1 |  |  |  |  |  |  |  |  |  | ***** | ***** | ***** | ***** | ***** | ***** | ***** | ***** | ***** | ***** | ***** | ***** |  | * |
|  |  | 2 |  |  |  |  |  |  |  |  |  | ***** | ***** | ***** | ***** | ***** | ***** | ***** | ***** | ***** | ***** | ***** | ***** |  | * |
|  |  | 3 |  |  |  |  |  |  |  |  |  | ***** | ***** | ***** | ***** | ***** | ***** | ***** | ***** | ***** | ***** | ***** | ***** |  | * |
|  | 2 | 1 |  |  |  |  |  |  |  |  |  | ***** | ***** | ***** | ***** | ***** | ***** | ***** | ***** | ***** | ***** | ***** | ***** |  | * |
|  |  | 2 |  |  |  |  |  |  |  |  |  | ***** | ***** | ***** | ***** | ***** | ***** | ***** | ***** | ***** | ***** | ***** | ***** |  | * |
|  |  | 3 |  |  |  |  |  |  |  |  |  | ***** | ***** | ***** | ***** | ***** | ***** | ***** | ***** | ***** | ***** | ***** | ***** |  | * |
| **1054** | 1 | 1 |  |  |  |  |  |  |  |  |  |  |  |  |  |  |  |  |  |  |  |  |  |  |  |
|  |  | 2 |  |  |  |  |  |  |  |  |  |  |  |  |  |  |  |  |  |  |  |  |  |  |  |
|  |  | 3 |  |  |  |  |  |  |  |  |  |  |  |  |  |  |  |  |  |  |  |  |  |  |  |
|  | 2 | 1 |  |  |  |  |  |  |  |  |  |  |  |  |  |  |  |  |  |  |  |  |  |  |  |
|  |  | 2 |  |  |  |  |  |  |  |  |  |  |  |  |  |  |  |  |  |  |  |  |  |  |  |
|  |  | 3 |  |  |  |  |  |  |  |  |  |  |  |  |  |  |  |  |  |  |  |  |  |  |  |
| **1056** | 1 | 1 |  |  |  |  |  |  |  |  |  |  |  |  |  |  |  |  |  |  |  |  |  |  |  |
|  |  | 2 |  |  |  |  |  |  |  |  |  |  |  |  |  |  |  |  |  |  |  |  |  |  |  |
|  |  | 3 |  |  |  |  |  |  |  |  |  |  |  |  |  |  |  |  |  |  |  |  |  |  |  |
|  | 2 | 1 |  |  |  |  |  |  |  |  |  |  |  |  |  |  |  |  |  |  |  |  |  |  |  |
|  |  | 2 |  |  |  |  |  |  |  |  |  |  |  |  |  |  |  |  |  |  |  |  |  |  |  |
|  |  | 3 |  |  |  |  |  |  |  |  |  |  |  |  |  |  |  |  |  |  |  |  |  |  |  |

ELISpot activities were positive if the median of peptide pools was >55 sfc/m and at least four times the median of the medium controls. Positive outcomes for each set are shown in gray; individual activities were combined to give a total CSP (C, light gray) or AMA1 (A, medium gray) response. * V=volunteer ID; T=time-point; A=assay number. The first three rows for each volunteer are the three assays for the first time-point, while the second three rows are the assays for the second time point. Missing samples are indicated by dots.
